# Correction: Rapid gain and loss of a chromosome drives key morphology and virulence phenotypes in the fungal pathogen *Histoplasma*

**DOI:** 10.1371/journal.pbio.3003651

**Published:** 2026-02-10

**Authors:** The PLOS Biology Staff

## Notice of Republication

This article was republished on January 28, 2026, to correct errors in Fig 5 and Fig 7 that were introduced during the typesetting process. The publisher apologizes for the errors. Please download this article again to view the correct version. The originally published, uncorrected article and the republished, corrected articles are provided here for reference.

## Supporting information

S1 File Originally published, uncorrected article. (PDF)

S2 File Republished, corrected article. (PDF)
