# Supplementary material for: Rapid gain and loss of a chromosome drives key morphology and virulence phenotypes in the fungal pathogen Histoplasma
Source: PLoS Biol. 2026 Jan 5;24(1):e3003224. doi: 10.1371/journal.pbio.3003224 (PMC12788632; doi:10.1371/journal.pbio.3003224)
Supplement: S1 Code — ZIP archive of PYTHON modules, scripts, and JUPYTER notebooks for analysis of the experiments in Fig 3. The code is documented in a README html file at the top level of the archive. (ZIP) [file pbio.3003224.s013.zip › time_course_analysis/notebooks/Figure_3.html]

Figure\_3


**Goal**: Merge time course sequencing and microscopy data into single table and generate plots.

In [1]:

```
%cd /home/mvoorhie/papers/SarahHeater_morphology/data_staging/
```

```
/home/mvoorhie/papers/SarahHeater_morphology/data_staging
```

In [2]:

```
from matplotlib import rcParams
# Note that family needs to be given before font
rcParams['font.family'] = 'sans-serif'
rcParams['font.sans-serif'] = 'Arial'
rcParams['svg.fonttype'] = 'none'
```

In [3]:

```
from MsvUtil import Table, hdict
import re
```

In [4]:

```
microscopy = Table.fromCsv("unblindedSHAS06MicroscopyScores.csv")
microscopy[0]
```

Out[4]:

```
blinded:		000_71b75f42325a.TIF
original:		SNAP-173231-0055_S22.tif
annotation:		5
```

In [5]:

```
sequencing = Table.fromCsv("PRJNA1257453_CNVs_and_allele_ratios.csv")
sequencing[0]
```

Out[5]:

```
sample:		25_D7_A__H_37C_S145
source:		A
initial_CNV:		smooth
initial_morphology:		H
condition:		37C
day:		7
CNV:		1.1025641025641026
oriented_allele_ratio:		0.0
```

In [6]:

```
len(microscopy)
```

Out[6]:

```
378
```

In [7]:

```
len(sequencing)
```

Out[7]:

```
102
```

In [8]:

```
tiff_re = re.compile(r"_(?P<sample>S[\d]+)\.tif$")
sample_to_microscopy = hdict(microscopy, lambda x: tiff_re.search(x["original"]).group("sample"))
len(sample_to_microscopy)
```

Out[8]:

```
83
```

In [9]:

```
sample_to_sequencing = dict(("S"+i["sample"].split("_")[0], i) for i in sequencing)
len(sample_to_sequencing)
```

Out[9]:

```
102
```

In [10]:

```
state_to_samples = {}
source_to_samples = {}
for i in sequencing:
    sample = "S"+i["sample"].split("_")[0]
    state = (i["initial_CNV"],i["initial_morphology"],i["condition"],i["day"])
    try:
        state_to_samples[state].append(sample)
    except KeyError:
        state_to_samples[state] = [sample]
    try:
        source_to_samples[i["source"]].append(sample)
    except KeyError:
        source_to_samples[i["source"]] = [sample]
len(state_to_samples),len(source_to_samples)
```

Out[10]:

```
(40, 8)
```

In [11]:

```
sample_to_mean = {}
for (sample, rows) in sample_to_microscopy.items():
    image_to_scores = hdict(rows, lambda x: x["original"])
    scores = []
    for (img, irows) in image_to_scores.items():
        score = irows[0]["annotation"]
        # Confirm that blinded scoring of repeated images was consistent
        for i in irows[1:]:
            assert(i["annotation"] == score)
        # Skip unscored images
        if(score != "None"):
            scores.append(int(score))
    # Confirm that all samples have at least 3 scored replicates
    assert(len(scores) >= 3)
    sample_to_mean[sample] = sum(int(i) for i in scores)/len(scores)
```

For the day 0 smooth:rough mixes, score as average of inputs

In [12]:

```
initial_mixes = (
("S5", ("S1","S4")),
("S6", ("S1","S4")),
("S7", ("S2","S3")),
("S8", ("S2","S3")),
("S13", ("S9","S12")),
("S14", ("S9","S12")),
("S15", ("S10","S11")),
("S16", ("S10","S11")),   
)
```

In [13]:

```
for (mix, inputs) in initial_mixes:
    assert(mix not in sample_to_mean)
    sample_to_mean[mix] = sum(sample_to_mean[i] for i in inputs)/2.
```

Aggregate

In [14]:

```
rows = []
for (sample, row) in sorted(sample_to_sequencing.items()):
    try:
        score = str(sample_to_mean[sample])
    except KeyError:
        assert(row["day"] == "14")
        score = ""
    rows.append(list(row)+[score])
table = Table(header = sequencing.header+["ImageMeans"], rows = rows)
```

In [15]:

```
table.writeTdt(open("S10_Table.txt","wt"))
```

In [16]:

```
sample_to_merge = dict(("S"+i["sample"].split("_")[0],i) for i in table)
```

In [17]:

```
table[0]
```

Out[17]:

```
sample:		1_D0_A__Y_first_S121
source:		A
initial_CNV:		smooth
initial_morphology:		Y
condition:		first
day:		0
CNV:		1.0526315789473684
oriented_allele_ratio:		0.0
ImageMeans:		1.0
```

In [18]:

```
%matplotlib nbagg
import matplotlib.pyplot as plt
```

In [19]:

```
import numpy as np
```

In [20]:

```
cs2 = {"A":"m","B":"g","C":'m',"D":"g",
       "E":"b","F":'b',"G":'b',"H":'b'}

import scipy.stats as stats
# define astrisks
def get_asterisks(p_value):
    if(np.isnan(p_value)):
        return ""
    elif p_value < 0.001:
        return "***"
    elif p_value < 0.01:
        return "**"
    elif p_value < 0.05:
        return "*"
    else:
        return ""
```

In [21]:

```
(fig,axes) = plt.subplots(nrows = 3, ncols = 2, figsize=(6, 5), sharex=True)

for (col, axrow) in zip(("CNV","oriented_allele_ratio","ImageMeans"),axes):
    for ((T,M),ax) in zip((("25C","Y"),("37C","H")), axrow):
        for (sources, color) in (
            ("AC","magenta"),
            ("BD","green"),
            ("EFGH","blue")):

            samples = []
            for source in sources:
                samples += source_to_samples[source]

            days = {}
            for i in samples:
                row = sample_to_merge[i]

                if((row["initial_morphology"] != M) or (not (row["condition"] in ("first",T)))):
                    continue

                day = int(row["day"])
                val = row[col]
                if(val == ""):
                    continue
                else:
                    val = float(val)
                try:
                    days[day].append(val)
                except KeyError:
                    days[day] = [val]

            x = sorted(days)
            y = np.array([np.mean(days[i]) for i in x])
            sd = np.array([np.std(days[i]) for i in x])
            ax.plot(x,y,color=color, marker = ".")
            ax.fill_between(x,y-sd,y+sd,color=color,alpha=.25)
            
            for n in range(len(x) - 1):
                t,p = stats.ttest_ind(days[x[n]],days[x[n+1]])
                sig = get_asterisks(p)
                if(sig != ""):
                    ax.text(x[n+1],y[n+1]+.05,sig,fontsize="medium",horizontalalignment="center",color=color)

axes[0,0].set_title("Yeast to Hyphae")
axes[0,1].set_title("Hyphae to Yeast")         
    
for ax in axes[2]:
    ax.set_xlabel("Days")
    
axes[0,0].set_ylabel("Chr7 Copy Number")
axes[1,0].set_ylabel("Fraction Aneuploid\nBarcode")
axes[2,0].set_ylabel("Morphology Score")
    
fig.tight_layout()
```
