# Supplementary material for: Rapid gain and loss of a chromosome drives key morphology and virulence phenotypes in the fungal pathogen Histoplasma
Source: PLoS Biol. 2026 Jan 5;24(1):e3003224. doi: 10.1371/journal.pbio.3003224 (PMC12788632; doi:10.1371/journal.pbio.3003224)
Supplement: S1 Code — ZIP archive of PYTHON modules, scripts, and JUPYTER notebooks for analysis of the experiments in Fig 3. The code is documented in a README html file at the top level of the archive. (ZIP) [file pbio.3003224.s013.zip › time_course_analysis/notebooks/Figure_3_SNP_table.html]

Figure\_3\_SNP\_table


**Goal**: Generate SNP table from the PRJNA1257851 whole genome sequencing samples in order to select two +/- aneuploidy pairs that are near isogenic but still distinguishable by some minimum number of SNPs.

Upstream steps to align reads to reference and generate basecounts matrix:

Align reads with BWA MEM:

```
for i in *_L00?_R1_001.fastq.gz; do
  RGROUP="${i%_L00?_R1_001.fastq.gz}";
  JOBNAME="${RGROUP}.SHRearrangedGenome1";
  bwa mem -t 20 -R "@RG\tID:${RGROUP}\tPL:ILLUMINA\tLB:${RGROUP}\tSM:${RGROUP}" SHRearrangedGenome1 "${i}" "${i/_R1_/_R2_}" 2> "${JOBNAME}.log" | samtools view -bS - > "${JOBNAME}.bam" && \
  samtools sort -o "${JOBNAME}.sorted.bam" "${JOBNAME}.bam" && \
  rm "${JOBNAME}.bam" && \
  samtools index "${JOBNAME}.sorted.bam"
done
```

Generate basecounts matrices in parallel *(samples can be regrouped at this stage if cores and/or RAM are limiting)*

```
for i in *.SHRearrangedGenome1.sorted.bam; do
  export SAMPLE="${i%.SHRearrangedGenome1.sorted.bam}";
  bam2basecounts.py -F SHRearrangedGenome1.fasta,SHRearrangedGenome1 "${SAMPLE}".hdf5 SHRearrangedGenome1 "${SAMPLE}" "${i}" &
done;
```

Combine individual runs to generate single basecounts file:

```
merge_basecounts.py -zo SHAS03_SHAS04_plus.basecounts.hdf5 *.hdf5
```

In [1]:

```
%cd ~/papers/SarahHeater_morphology/data_staging/
```

```
/home/mvoorhie/papers/SarahHeater_morphology/data_staging
```

In [2]:

```
import h5py
import numpy as np
```

In [3]:

```
from Collisions import RefCollisions, SortLoci
from Gff3 import Gff3file
from Locus import Locus
from MsvUtil import Table, hdict, revdict
from RepeatClassification import AnnealedRepeatClassification
from SNPTable import GenomicBiallelicSNPTable
from UnionedGenomeFactory import FastaGenomeFactory
```

In [4]:

```
f = FastaGenomeFactory(
    open("SHRearrangedGenome1.fasta"),
    "SHRearrangedGenome1",
    "SHRearrangedGenome1.genes.gff3")

genome = f.getGenome("SHRearrangedGenome1")

raw = Gff3file.fromFile(open("SHRearrangedGenome1.ltrharvest.default.gff3","rt"))
LTR_repeats = {"SHRearrangedGenome1":raw.type_to_features["repeat_region"]}
raw = Gff3file.fromFile(open("SHRearrangedGenome1.Maggy.gff3","rt"))
maggy_orfs = {"SHRearrangedGenome1":raw.type_to_features["MAGGY"]}

arc = {genome:AnnealedRepeatClassification(genome, maggy_orfs, LTR_repeats)}
```

In [5]:

```
basecounts_hdf5 = h5py.File("SHAS03_SHAS04_plus.basecounts.hdf5","r")
```

In [6]:

```
samples = [
 # SHAS03 dup derived (A/B/C)
 # SHAS03 "A" progeny
 b'A12Rough_yesD_13_322_S40_L001',
 b'A12Smooth_yesD_9_118_S16_L001',
 b'A20Smooth_yesD_10_119_S17_L001',
 b'A23Rough_yesD_14_123_S20_L001',
 b'A23Smooth_yesD_11_120_S18_L001',

 # SHAS03 "B" progeny
 b'B12Rough_yesD_26_134_S31_L001',
 b'B12Smooth_yesD_20_129_S26_L001',
 b'B13Rough_yesD_27_135_S32_L001',
 b'B15Smooth_yesD_22_131_S28_L001',
 b'B2Rough_yesD_24_132_S29_L001',
 b'B2Smooth_yesD_15_124_S21_L001',
 b'B3Rough_yesD_25_133_S30_L001',
 b'B3Smooth_yesD_16_125_S22_L001',
 b'B4Smooth_yesD_17_126_S23_L001',
 b'B5Smooth_yesD_18_127_S24_L001',
 b'B6Smooth_yesD_19_128_S25_L001',

 # SHAS03 "C" progeny
 b'C15Rough_yesD_31_139_S36_L001',
 b'C15Smooth_yesD_29_137_S34_L001',
 b'C19Rough_yesD_32_140_S37_L001',
 b'C19Smooth_yesD_30_138_S35_L001',
 b'C2Smooth_yesD_28_136_S33_L001',

 # SHAS03 nodup derived (X/Y/Z)
 b'X11Rough_NOD_5_315_S39_L001',
 b'X11Smooth_NOD_1_111_S11_L001',
 b'Y2Smooth_NOD_2_112_S12_L001',
 b'Z18Rough_NOD_8_117_S15_L001',
 b'Z18Smooth_NOD_4_314_S38_L001',
 b'Z1Smooth_NOD_3_113_S13_L001', 

 # SHAS04
 b'11_A4_Smooth_S195',
 b'12_A16_Smooth_S196',
 b'13_B8_Smooth_S197',
 b'14_A4_Rough_S198',
 b'15_A11_Rough_S199',
 b'16_A12_Rough_S200',
 b'17_A16_Rough_S201',
 b'18_B8_Rough_S202',
]
```

In [7]:

```
%%time
SNPs1 = GenomicBiallelicSNPTable.fromBaseCountsHDF5(basecounts_hdf5, samples = samples)
```

```
CPU times: user 6min 51s, sys: 37.4 s, total: 7min 28s
Wall time: 7min 28s
```

In [8]:

```
t = 0
for (contig,table) in SNPs1.contig2table.items():
    print(contig, table.Bmaj.shape)
    t += len(table.positions)
t
```

```
CM029948 (0,)
ChromSixTwo (1, 35)
ChromTwoSix (18, 35)
JAEVHH010000002 (5, 35)
JAEVHH010000003 (14, 35)
JAEVHH010000004 (21, 35)
JAEVHH010000005 (3, 35)
JAEVHH010000007 (3, 35)
JAEVHH010000009 (0,)
JAEVHH010000010 (0,)
JAEVHH010000011 (0,)
JAEVHH010000012 (0,)
```

Out[8]:

```
65
```

In [9]:

```
%%time
SNPs1.toHDF5("Barcoded_pairs_1.SNPs1.hdf5")
```

```
CPU times: user 16.2 ms, sys: 4.02 ms, total: 20.3 ms
Wall time: 20.2 ms
```
