# Supplementary material for: Rapid gain and loss of a chromosome drives key morphology and virulence phenotypes in the fungal pathogen Histoplasma
Source: PLoS Biol. 2026 Jan 5;24(1):e3003224. doi: 10.1371/journal.pbio.3003224 (PMC12788632; doi:10.1371/journal.pbio.3003224)
Supplement: S1 Code — ZIP archive of PYTHON modules, scripts, and JUPYTER notebooks for analysis of the experiments in Fig 3. The code is documented in a README html file at the top level of the archive. (ZIP) [file pbio.3003224.s013.zip › time_course_analysis/notebooks/Figure_3_allele_ratios.html]

Figure\_3\_allele\_ratios


**Goal**: Calculate allele ratios at diagnostic positions for the samples in PRJNA1257453 so that we can attribute CNV changes in the critical region to either selection or spontaneous conversion.

- Specifically, the starting strains for this experiment were A23 smooth/rough and X11 smooth/rough

Upstream steps to align reads to reference and generate basecounts matrix:

Align reads with BWA MEM:

```
for i in *_L00?_R1_001.fastq.gz; do
  RGROUP="${i%_L00?_R1_001.fastq.gz}";
  JOBNAME="${RGROUP}.SHRearrangedGenome1";
  bwa mem -t 20 -R "@RG\tID:${RGROUP}\tPL:ILLUMINA\tLB:${RGROUP}\tSM:${RGROUP}" SHRearrangedGenome1 "${i}" "${i/_R1_/_R2_}" 2> "${JOBNAME}.log" | samtools view -bS - > "${JOBNAME}.bam" && \
  samtools sort -o "${JOBNAME}.sorted.bam" "${JOBNAME}.bam" && \
  rm "${JOBNAME}.bam" && \
  samtools index "${JOBNAME}.sorted.bam"
done
```

Generate basecounts matrices in parallel *(samples can be regrouped at this stage if cores and/or RAM are limiting)*

```
for i in *.SHRearrangedGenome1.sorted.bam; do
  export SAMPLE="${i%.SHRearrangedGenome1.sorted.bam}";
  bam2basecounts.py -F SHRearrangedGenome1.fasta,SHRearrangedGenome1 "${SAMPLE}".hdf5 SHRearrangedGenome1 "${SAMPLE}" "${i}" &
done;
```

Combine individual runs to generate single basecounts file:

```
merge_basecounts.py -zo PRJNA1257453.basecounts.hdf5 *.hdf5
```

Coverage hdf5 files to estimate CNV changes in the critical region were generated from BWA MEM BAM files (generated as above) followed by:

```
hdf5cov.py -sz -F SHRearrangedGenome1.fasta,SHRearrangedGenome1 \
  $(for i in *.SHRearrangedGenome1.sorted.bam; do echo -n "${i%.SHRearrangedGenome1.sorted.bam} ${i} "; done;);
done;
```

Additionally, the same reads were aligned to a draft assembly of the G217B-derived UCSF3 strain (GCA\_051313235.1) which corrects for two chromosomal rearrangements unique to the previously Nanopore sequenced G217B-derived UCSF1 strain (GCA\_017607445.1). Both genome models gave similar results for the CNV calculation. The CNV results based on the newer (UCSF3) model are reported in S10\_Table.txt are figure 3 of the paper.

As the SNP analysis uses the genome model only as an alignment reference and infers all allele frequencies only from the aligned Illumina reads, it should be robust to the structural differences between the two genome models and was not repeated with the newer assembly.

In [1]:

```
%cd ~/papers/SarahHeater_morphology/data_staging/
```

```
/home/mvoorhie/papers/SarahHeater_morphology/data_staging
```

In [2]:

```
%matplotlib nbagg
import matplotlib.pyplot as plt
```

In [3]:

```
from csv import writer
import h5py
import numpy as np
import re
```

In [4]:

```
from Collisions import RefCollisions, SortLoci, LocusDiff
from Gff3 import Gff3file
from Locus import Locus
from MsvUtil import Table, hdict, revdict
from RepeatClassification import AnnealedRepeatClassification
from SNPTable import GenomicBiallelicSNPTable
from SNP_tools import SNP
from UnionedGenomeFactory import FastaGenomeFactory
```

# SNP "barcode" analysis¶

This analysis infers proportions of the original strains at each timepoint based on allele ratios of strain-unique SNPs.

Re-derive previously identified diagnositc SNPs and cut basecounts matrix to those positions.

In [5]:

```
f = FastaGenomeFactory(
    open("SHRearrangedGenome1.fasta"),
    "SHRearrangedGenome1",
    "SHRearrangedGenome1.genes.gff3")

genome = f.getGenome("SHRearrangedGenome1")

raw = Gff3file.fromFile(open("SHRearrangedGenome1.ltrharvest.default.gff3","rt"))
LTR_repeats = {"SHRearrangedGenome1":raw.type_to_features["repeat_region"]}
raw = Gff3file.fromFile(open("SHRearrangedGenome1.Maggy.gff3","rt"))
maggy_orfs = {"SHRearrangedGenome1":raw.type_to_features["MAGGY"]}

arc = {genome:AnnealedRepeatClassification(genome, maggy_orfs, LTR_repeats)}
```

Load SNP table based on analysis of earlier sequencing experiments, which identified neutral SNPs diagnostic for the strains chosen for this experiment.

In [6]:

```
SNPs1 = GenomicBiallelicSNPTable.fromHDF5("Barcoded_pairs_1.SNPs1.hdf5")
```

Cut to non-transposon SNPs

In [7]:

```
SNPs3 = SNPs1.remove_loci(arc[genome].annealed_loci)
t = 0
for (contig,table) in SNPs3.contig2table.items():
    print(contig, table.Bmaj.shape)
    t += len(table.positions)
t
```

```
ChromSixTwo (0, 35)
ChromTwoSix (1, 35)
JAEVHH010000002 (1, 35)
JAEVHH010000003 (2, 35)
JAEVHH010000004 (1, 35)
JAEVHH010000005 (1, 35)
JAEVHH010000007 (1, 35)
```

Out[7]:

```
7
```

Load positional allele frequencies

In [8]:

```
basecounts = h5py.File("PRJNA1257453.basecounts.hdf5","r")
```

In [9]:

```
alphabet = "".join(i.decode() for i in basecounts["alphabet"])
alphabet
```

Out[9]:

```
'ACGTN'
```

In [10]:

```
snps = []
a = 0
b = 1
S = []
for contig in SNPs3.corder:
    table = SNPs3.contig2table[contig]
    for ((p,i),s) in zip(table, table.alleles.T):
        print(contig,p,alphabet[s[a]],alphabet[s[b]])
        print("".join(alphabet[s[j]] for j in i))
        snps.append(SNP({"REF":alphabet[s[a]],"ALT":alphabet[s[b]]},
                        "NA",Locus(contig,p,p,"+",genome)))
        print(snps[-1])
        S.append(i)
S = np.array(S)
```

```
ChromTwoSix 2072721 C A
AAAAACCCCCCCCCCCAAAAAAAAAAAAAAAAAAA
SNP: ChromTwoSix:2072721..2072721 (+) C->A (NA)
JAEVHH010000002 2082378 A G
GGGGGGGGGGGGGGGGGGGAGGGGGGGGGGGGGGG
SNP: JAEVHH010000002:2082378..2082378 (+) A->G (NA)
JAEVHH010000003 1006867 A C
CCCCCCCCCCCCCCCCCCCCCCCCCCCCCCCCCCA
SNP: JAEVHH010000003:1006867..1006867 (+) A->C (NA)
JAEVHH010000003 4582209 T C
CCCCCCCCCCCCCCCCCCCCCCCCCCCCTCCCCCC
SNP: JAEVHH010000003:4582209..4582209 (+) T->C (NA)
JAEVHH010000004 1752899 T C
CCCCCCCCCCCCCCCCCCCCCTTTTTTCCCCCCCC
SNP: JAEVHH010000004:1752899..1752899 (+) T->C (NA)
JAEVHH010000005 3366448 A G
GGGGGGGAAGGGGGGGGGGGGGGGGGGGGGGGGGG
SNP: JAEVHH010000005:3366448..3366448 (+) A->G (NA)
JAEVHH010000007 1296086 C G
GGGGGGGGGGGGGGGGGGGGGCCCCCCGGGGGGGG
SNP: JAEVHH010000007:1296086..1296086 (+) C->G (NA)
```

In [11]:

```
SNPs_bc = np.concatenate([
    basecounts["/SHRearrangedGenome1/%s" % snp.Locus().ref][:,snp.Locus().start:snp.Locus().start+1,:]
    for snp in snps],axis=1)
SNPs_bc.shape
```

Out[11]:

```
(102, 7, 5)
```

Calculate allele ratios

In [12]:

```
alpha2i = dict((c,n) for (n,c) in enumerate(alphabet))
```

In [13]:

```
allele_ratios = []
for (n, snp) in enumerate(snps):
    sbc = SNPs_bc[:,n,:]
    print(snp)
    a = sbc[:,alpha2i[snp.ref]].astype("float")
    b = sbc[:,alpha2i[snp.alt]].astype("float")
    # orient on minor allele
    if(sum(a) > sum(b)):
        (a,b) = (b,a)
    allele_ratios.append(a/(a+b))
allele_ratios = np.array(allele_ratios)
allele_ratios.shape, allele_ratios.dtype
```

```
SNP: ChromTwoSix:2072721..2072721 (+) C->A (NA)
SNP: JAEVHH010000002:2082378..2082378 (+) A->G (NA)
SNP: JAEVHH010000003:1006867..1006867 (+) A->C (NA)
SNP: JAEVHH010000003:4582209..4582209 (+) T->C (NA)
SNP: JAEVHH010000004:1752899..1752899 (+) T->C (NA)
SNP: JAEVHH010000005:3366448..3366448 (+) A->G (NA)
SNP: JAEVHH010000007:1296086..1296086 (+) C->G (NA)
```

Out[13]:

```
((7, 102), dtype('float64'))
```

In [14]:

```
sample_re = re.compile(r"^(?P<n>[\d]+)_"
                       r"D(?P<day>[\d]+)_"
                       r"(?P<source>[A-H])__"
                       r"(?P<m1>.*)_"
                       r"(?P<condition>37C|first|25C)_"
                       r"(?P<run>S[\d]+)$")

class SHAS06_sample:
    @classmethod
    def fromName(cls, name):
        return cls(**sample_re.search(name).groupdict())
    def __init__(self, n, day, source, m1, condition, run):
        self.n = int(n)
        self.day = int(day)
        self.source = source
        self.m1 = m1
        self.condition = condition
        self.run = run
    def starting_genotype(self):
        if(self.source in "AC"):
            return "smooth"
        elif(self.source in "BD"):
            return "rough"
        else:
            return "mix"
    # Unique identifier
    def state(self):
        return (self.source,self.m1,self.condition,self.day)
    @property
    def name(self):
        return "%d_D%d_%s__%s_%s_%s" % (
        self.n,self.day,self.source,self.m1,self.condition,self.run)
    @property
    def bam(self):
        return self.name+".SHRearrangedGenome1.sorted.bam"
    @property
    def bam2(self):
        return self.name+".G217B_ATCC.V0.05.sorted.bam"
```

In [15]:

```
samples = [SHAS06_sample.fromName(i.decode()) for i in basecounts["samples"]]
len(samples)
```

Out[15]:

```
102
```

In [16]:

```
prefix2sample = dict((i.name,i) for i in samples)
sample2col = {}
for (n,i) in enumerate(basecounts["samples"]):
    try:
        sample = prefix2sample[i.decode()]
        sample2col[sample] = n
    except KeyError:
        continue
len(sample2col)
```

Out[16]:

```
102
```

In [17]:

```
estimated_mix = np.mean(allele_ratios[(4,6),:],axis=0)
```

estimated\_mix needs to be oriented so that 0 always corresponds to the no dup (smooth) parental)

In [18]:

```
source2flip = {
"A": False, #("A23 Smooth",),
"B": True, #("A23 Rough",),
"C": True, #("X11 Smooth",),
"D": False, #("X11 Rough",),
"E": False, #("A23 Smooth","X11 Rough",),
"F": False, #("A23 Smooth","X11 Rough",),
"G": True, #("X11 Smooth","A23 Rough",),
"H": True, #("X11 Smooth","A23 Rough",),
}
```

In [19]:

```
oriented_mix = estimated_mix[:]
for (sample,col) in sample2col.items():
    if(source2flip[sample.source]):
        oriented_mix[col] = 1-oriented_mix[col]
```

# CNV analysis¶

This analysis estimates copy number of the critical region relative to a single copy normalizer region for each timepoint.

## Using UCSF1-based genome model¶

Define non-transposon locations in the critical region

In [20]:

```
# Find the locations not in the arcs
garc = arc[genome]
NotGarc = {}

for (name,contig) in genome.Contigs():
    locus = contig.Locus()
    NotGarc[name] = LocusDiff(locus, garc.annealed_loci)

NotGarc["YesGarc"] = garc.annealed_loci

# Add the Critical region to this NotGarc List

CriticalLocus = Locus("ChromSixTwo",1410000,1789999,"+",genome)

NotGarc["CriticalLocus"] = LocusDiff(CriticalLocus, garc.annealed_loci)
# what this gives is ChromSixTwo:1516286..1789999

critical = NotGarc["CriticalLocus"][0]
```

Define known 1N normalizer region

In [21]:

```
norm_locus = Locus("JAEVHH010000004",2528478,3741559,"+",genome)
norm_locus
```

Out[21]:

```
JAEVHH010000004:2528478..3741559 (+)
```

In [22]:

```
hdf1 = h5py.File("SHAS06.coverage.hdf5","r")
```

Calculate the CNV estimate as the median of the critical region coverage relative to normalizer region coverage for each sample

In [23]:

```
sample_norm = {}
gname = "SHRearrangedGenome1"

for sample in samples:
    Y = np.array(hdf1["/%s/%s/%s/L/+/" % (
        gname,norm_locus.ref,sample.bam)][norm_locus.start:norm_locus.stop+1])
    for (side,strand) in (("L","-"),("R","+"),("R","-")):
        Y += np.array(hdf1["/%s/%s/%s/%s/%s/" % 
                      (gname,norm_locus.ref,sample.bam,side,strand)][norm_locus.start:norm_locus.stop+1])
    sample_norm[sample] = np.median(Y)
```

In [24]:

```
CNV = {}

for sample in samples:
    Y = np.array(hdf1["/%s/%s/%s/L/+/" % (gname,critical.ref,sample.bam)][critical.start:critical.stop+1])
    for (side,strand) in (("L","-"),("R","+"),("R","-")):
        Y += np.array(hdf1["/%s/%s/%s/%s/%s/" % 
                      (gname,critical.ref,sample.bam,side,strand)][critical.start:critical.stop+1])
    CNV[sample] = np.median(Y)/sample_norm[sample]
```

In [25]:

```
def CNV_to_ratio(x):
    return min(1,max(0,x - 1))
```

In [26]:

```
CNV_vector = np.zeros_like(estimated_mix)
for (sample, val) in CNV.items():
    CNV_vector[sample2col[sample]] = val
```

## Using UCSF1-based genome model¶

Define critical region relative to transposon-rich boundaries

In [27]:

```
# Find the locations not in the arcs
garc = arc[genome]
NotGarc = {}

for (name,contig) in genome.Contigs():
    locus = contig.Locus()
    NotGarc[name] = LocusDiff(locus, garc.annealed_loci)

NotGarc["YesGarc"] = garc.annealed_loci

# Add the Critical region to this NotGarc List

CriticalLocus = Locus("ChromSixTwo",1410000,1789999,"+",genome)

NotGarc["CriticalLocus"] = LocusDiff(CriticalLocus, garc.annealed_loci)
# what this gives is ChromSixTwo:1516286..1789999

critical = NotGarc["CriticalLocus"][0]
```

Define known 1N normalizer region

In [28]:

```
norm_locus = Locus("JAEVHH010000004",2528478,3741559,"+",genome)
norm_locus
```

Out[28]:

```
JAEVHH010000004:2528478..3741559 (+)
```

In [29]:

```
hdf1 = h5py.File("SHAS06.coverage.hdf5","r")
```

Calculate the CNV estimate as the median of the critical region coverage relative to normalizer region coverage for each sample

In [30]:

```
sample_norm = {}
gname = "SHRearrangedGenome1"

for sample in samples:
    Y = np.array(hdf1["/%s/%s/%s/L/+/" % (
        gname,norm_locus.ref,sample.bam)][norm_locus.start:norm_locus.stop+1])
    for (side,strand) in (("L","-"),("R","+"),("R","-")):
        Y += np.array(hdf1["/%s/%s/%s/%s/%s/" % 
                      (gname,norm_locus.ref,sample.bam,side,strand)][norm_locus.start:norm_locus.stop+1])
    sample_norm[sample] = np.median(Y)
```

In [31]:

```
CNV = {}

for sample in samples:
    Y = np.array(hdf1["/%s/%s/%s/L/+/" % (gname,critical.ref,sample.bam)][critical.start:critical.stop+1])
    for (side,strand) in (("L","-"),("R","+"),("R","-")):
        Y += np.array(hdf1["/%s/%s/%s/%s/%s/" % 
                      (gname,critical.ref,sample.bam,side,strand)][critical.start:critical.stop+1])
    CNV[sample] = np.median(Y)/sample_norm[sample]
```

In [32]:

```
def CNV_to_ratio(x):
    return min(1,max(0,x - 1))
```

In [33]:

```
CNV_vector_0 = np.zeros_like(estimated_mix)
for (sample, val) in CNV.items():
    CNV_vector_0[sample2col[sample]] = val
```

## Using draft UCSF3 genome model¶

In [34]:

```
f = FastaGenomeFactory(
    open("G217B_ATCC.V0.05.fasta"),
    "G217B_ATCC.V0.05",
    "G217B_ATCC.V0.05.BLAT.gff3")

genome = f.getGenome("G217B_ATCC.V0.05")

raw = Gff3file.fromFile(open("G217B_ATCC.V0.05.ltrharvest.default.gff3","rt"))
LTR_repeats = {"G217B_ATCC.V0.05":raw.type_to_features["repeat_region"]}
raw = Gff3file.fromFile(open("G217B_ATCC.V0.05.Maggy.gff3","rt"))
maggy_orfs = {"G217B_ATCC.V0.05":raw.type_to_features["MAGGY"]}

arc = {genome:AnnealedRepeatClassification(genome, maggy_orfs, LTR_repeats)}
```

Define CNV region as portions of UCSF3 chr7 outside of transposon blocks

In [35]:

```
# Find the locations not in the arcs
garc = arc[genome]
NotGarc = {}

for (name,contig) in genome.Contigs():
    locus = contig.Locus()
    NotGarc[name] = LocusDiff(locus, garc.annealed_loci)

NotGarc["YesGarc"] = garc.annealed_loci

chr7_not_transposon = NotGarc["chr7"]
```

Define known 1N normalizer region

In [36]:

```
norm_locus = Locus("chr3",2533970,3746857,"+",genome)
norm_locus
```

Out[36]:

```
chr3:2533970..3746857 (+)
```

In [37]:

```
hdf1 = h5py.File("SHAS06.G217B_ATCC.V0.05.coverage.hdf5","r")
```

Calculate the CNV estimate as the median of the critical region coverage relative to normalizer region coverage for each sample

In [38]:

```
sample_norm = {}
gname = "G217B_ATCC.V0.05"

for sample in samples:
    Y = np.array(hdf1["/%s/%s/%s/L/+/" % (
        gname,norm_locus.ref,sample.bam2)][norm_locus.start:norm_locus.stop+1])
    for (side,strand) in (("L","-"),("R","+"),("R","-")):
        Y += np.array(hdf1["/%s/%s/%s/%s/%s/" % 
                      (gname,norm_locus.ref,sample.bam2,side,strand)][norm_locus.start:norm_locus.stop+1])
    sample_norm[sample] = np.median(Y)
```

In [39]:

```
CNV = {}

for sample in samples:
    Y = []
    for region in chr7_not_transposon:
        Y_region = np.array(hdf1["/%s/chr7/%s/L/+/" % (gname,sample.bam2)][region.start:region.stop+1])
        for (side,strand) in (("L","-"),("R","+"),("R","-")):
            Y_region += np.array(hdf1["/%s/chr7/%s/%s/%s/" % 
                          (gname,sample.bam2,side,strand)][region.start:region.stop+1])
        Y.append(Y_region)
    Y = np.concatenate(Y)
    CNV[sample] = np.median(Y)/sample_norm[sample]
```

In [40]:

```
CNV_vector = np.zeros_like(estimated_mix)
for (sample, val) in CNV.items():
    CNV_vector[sample2col[sample]] = val
```

Confirm that CNV result is robust to genome model:

In [41]:

```
fig = plt.figure()
plt.plot(CNV_vector, CNV_vector_0, "k.")
```

Out[41]:

```
[<matplotlib.lines.Line2D at 0x7fc5a66e05e0>]
```

Write allele ratios and CNV estimates to table

In [42]:

```
out = writer(open("PRJNA1257453_CNVs_and_allele_ratios.csv","wt"))
out.writerow(("sample","source","initial_CNV","initial_morphology","condition","day", "CNV","oriented_allele_ratio"))
for (sample,col) in sorted(sample2col.items(), key = lambda x: (x[0].state(), x[0].name)):
    out.writerow((sample.name,
                  sample.source,
                  sample.starting_genotype(),
                  sample.m1,
                  sample.condition,
                  sample.day,
                  CNV_vector[col],
                  oriented_mix[col]))
del out
```
