# Supplementary material for: Rapid gain and loss of a chromosome drives key morphology and virulence phenotypes in the fungal pathogen Histoplasma
Source: PLoS Biol. 2026 Jan 5;24(1):e3003224. doi: 10.1371/journal.pbio.3003224 (PMC12788632; doi:10.1371/journal.pbio.3003224)
Supplement: S1 Code — ZIP archive of PYTHON modules, scripts, and JUPYTER notebooks for analysis of the experiments in Fig 3. The code is documented in a README html file at the top level of the archive. (ZIP) [file pbio.3003224.s013.zip › time_course_analysis/README.html]

README


This file documents the computational methods for the time
course analysis in the paper "Rapid gain and loss of a
chromosome drives key morphology and virulence phenotypes
in *Histoplasma*, a fungal pathogen of humans"

The following Jupyter
notebooks document the analysis:

1. Figure\_3\_SNP\_table.ipynb
   generates a SNP table of previously sequenced G217B-derived
   strains. This table is used in subsequent analysis and was used
   to select otherwise isogenic strains that differed by a few
   non-genic "barcode" SNPs and by chr7 CNV to use as input for the
   time course experiment.- Figure\_3\_allele\_ratios.ipynb
     analyzes aligned Illumina reads from each time point to infer
     proportions of the input strains from the allele ratios at the
     "barcode" SNPs and calculates the CNV of chr7 relative to a
     normalizer region.- Figure\_3.ipynb
       collates the allele ratios and CNV values from the sequence
       analysis with morphological scores from microsopy of each
       timepoint, generating S10\_table.txt. It then uses this table to
       generate the plots for Figure 3.

Upstream batch processing steps and sequence accessions are
described in the notebooks.

This archive is organized as:

notebooks/: ipynb files and static html exports of the above notebooks. bin/: upstream batch processing scripts lib/: python3 modules required by the scripts and/or notebooks

The above code is released under the terms of the MIT license,
which is included in the COPYING file in the root of this
archive.

This pipeline depends on the BWA MEM aligner and on the
following external python3 modules:

- h5py- matplotlib- numpy- scipy

The analysis was carried out on
a Debian 11 server with a 20
core Xeon E5-2640 v4 processor and 64 GB of RAM. Much of the code
has also been tested on Debian 12
and Ubuntu 24.04. For these and
related Linux distributions, the dependencies can be installed
via:

```
      apt-get install python3 jupyter bwa python3-matplotlib python3-h5py python3-numpy python3-scipy
```

For additional information, please contact Mark Voorhies (mark.voorhies@ucsf.edu)
