# Supplementary material for: Correction: Rapid gain and loss of a chromosome drives key morphology and virulence phenotypes in the fungal pathogen Histoplasma
Source: PLoS Biol. 2026 Feb 10;24(2):e3003651. doi: 10.1371/journal.pbio.3003651 (PMC12890133; doi:10.1371/journal.pbio.3003651)
Supplement: S1 File — (PDF) [file pbio.3003651.s001.pdf]

PLOS Biology

RESEARCH ARTICLE

Rapid gain and loss of a chromosome drives key morphology and virulence phenotypes in the fungal pathogen *Histoplasma*

Sarah Heater[1], Mark Voorhies[1], Rosa A. Rodriguez[1], Bevin C. English[1,2], Anita Sil[1,3]*

1 Department of Microbiology and Immunology, University of California San Francisco, San Francisco, California, United States of America, **2** CoLabs, University of California San Francisco, San Francisco, California, United States of America, **3** Biohub—San Francisco, San Francisco, California, United States of America

* anita.sil@ucsf.edu

Check for updates

OPEN ACCESS

Citation: Heater S, Voorhies M, Rodriguez RA, English BC, Sil A (2026) Rapid gain and loss of a chromosome drives key morphology and virulence phenotypes in the fungal pathogen *Histoplasma*. PLoS Biol 24(1): e3003224. https://doi.org/10.1371/journal.pbio.3003224

Academic Editor: Aaron P. Mitchell, University of Georgia, UNITED STATES OF AMERICA

Received: May 16, 2025

Accepted: December 12, 2025

Published: January 5, 2026

Copyright: © 2026 Heater et al. This is an open access article distributed under the terms of the Creative Commons Attribution License, which permits unrestricted use, distribution, and reproduction in any medium, provided the original author and source are credited.

Data availability statement: DNA and RNA sequencing reads were submitted to GenBank under BioProjects PRJNA1256922, PRJNA1257453, PRJNA1257851, PRJNA1259587, PRJNA1283525, and GEO series GSE296938. Genome assemblies from BioProjects PRJNA682645 and

Abstract

Heritable phenotypic switches are fundamental to the ability of cells to respond to specific conditions. Such switches are key to the success of environmental pathogens, which encounter disparate conditions as they transition between the environment and host. We determine that the copy number of chromosome seven in the thermally dimorphic fungus *Histoplasma* dramatically affects the rate of transition. Though *Histoplasma* is haploid, a second copy of this chromosome is present in natural isolates of multiple *Histoplasma* species and is gained and lost at a high rate. Cells carrying two copies of this chromosome exhibit aspects of the environmental transcriptome even under host-like conditions and have a competitive advantage in the transition to the environmental form. Conversely, these cells are considerably less virulent than euploid cells and have a competitive disadvantage in the mouse model of infection. Chromosome seven contains a previously unstudied transcription factor that, when expressed at higher copy number in euploid *Histoplasma*, is sufficient to promote some of the key phenotypes of aneuploidy. We hypothesize that rapid gain and loss of this chromosome benefits *Histoplasma* by increasing phenotypic variation, thus helping populations of cells survive abrupt transitions between environment and host.

Introduction

Genetic diversity within a population can enable survival, since a portion of the population may survive conditions lethal to most of the community. Genome instability is one mechanism by which a community can rapidly diversify, preparing it to survive subsequent fluctuations. One form of genome instability is copy number variation (CNV), ranging from changes in copy number of the entire genome, a particular chromosome, or a specific gene. CNV has been associated with improving fungal fitness

PRJNA1196486 were used. Previously published sequencing of natural isolates from BioProjects PRJNA416769, PRJNA868688, and PRJNA1003095 were also used for analysis. Accessions for data sets used in Fig 7G are given in S5 Table. S1 Table lists strains used in this study. S1 Code provides custom code used in this study.

Funding: We acknowledge NIH R37AI066224 (to AS), NIH 1R01AI172258-01A1 (to AS), an Achievement Rewards for College Scientists (ARCS) Scholarship (to SH) and funding from T32AI060537 (to SH). AS is a Biohub, San Francisco, investigator. The funders did not play any role in the study design, data collection and analysis, decision to publish, or preparation of the manuscript.

Competing interests: The authors have declared that no competing interests exist.

Abbreviations: CNV, copy number variation; HMM, *Histoplasma*-macrophage medium; HPS, hyphal-phase specific transcripts; HYF, Hyphae Promoting Factor; PK, protein kinase; SHP, small hyphal peptide; TFs, transcription factors; YPS, yeast-phase specific transcripts.

in a wide range of stress conditions, including exposure to antifungals, high temperature, and mammalian infection [1–6].

Exposure to extreme fluctuations is fundamental to the lifecycle of environmental pathogens such as *Histoplasma* species, which transition between the environment and the mammalian host. *Histoplasma* grows as sporogenous hyphae in soil at environmental temperatures. When aerosolized and inhaled by a human or other mammal, *Histoplasma* transitions to growth as pathogenic yeast in response to body temperature. Upon return to the environment, potentially via excretion in guano [7] or after host death [8], *Histoplasma* transitions back to its hyphal form. In vitro, simply shifting the temperature from ambient room temperature to body temperature is sufficient to trigger this morphology transition. Understanding the molecular basis of morphologic transitions in response to temperature is fundamental to understanding *Histoplasma* pathogenesis.

Histoplasma is considered a high priority pathogen by WHO and is found globally, with specific regions of hyperendemicity [9,10] where most of the human population may be exposed to *Histoplasma* within their lifetimes [11,12]. A recent analysis suggested that *Histoplasma* incidence among people with AIDS in Latin America may be similar to that of tuberculosis [13]. Despite this high prevalence, infections are often misdiagnosed and it is considered a neglected disease [13].

General differences between *Histoplasma* species in terms of human health and pathogenicity are unknown. Comparisons between individual isolates of multiple *Histoplasma* species have shown phenotypic variation and differences in immune response [14,15]. *H. ohiense* and *H. mississippiense* are proposed *Histoplasma* species found in North America that have historically been most sequenced. Interestingly, as this study highlights, there can be large variance in characteristics including pathogenicity among different strains of one *Histoplasma* species.

Developmental transitions driving *Histoplasma* towards the pathogenic yeast or the environmental hyphal morphology in response to temperature are key characteristics of this fungus. Recently, some progress has been made in our understanding of the molecular mechanisms underlying these transitions, including the discovery of several transcription factors (TFs) that drive each morphology [16–21]. However, one difficulty in these investigations has been a lack of consistency in these transitions in vitro. While temperature is sufficient to trigger transitions between yeast and hyphal growth in the laboratory, the rate at which this transition occurs has not been fully consistent for unknown reasons. Differences in the rate of transition were previously misattributed to the presence or absence of the gene *MSB2* [22,23].

Enabled by our recent whole genome assembly [24], we found that frequent gain and loss of a specific chromosome in this haploid organism affects the rate of hyphal formation in response to temperature shift. Aneuploid *Histoplasma*, carrying an additional copy of chromosome 7, was biased towards growth as hyphae and rapidly transitions from yeast to hyphae in response to ambient temperature. Conversely, euploid *Histoplasma* was biased towards growth as yeast and underwent a much slower transition to hyphae in response to temperature. We discovered that copy number of this chromosome varies in many natural and laboratory

isolates, and that this aneuploidy was gained and lost at a high rate. Cells carrying two copies of chromosome 7 were significantly less virulent in the mouse model of infection than cells carrying a single copy. A TF on chromosome 7 was sufficient to drive some of the phenotypes associated with an extra copy of chromosome 7, including transcriptional upregulation of a conserved set of genes associated with the hyphal morphology. This work improves our understanding of how variation within a population affects the ability of this important fungal pathogen of humans to thrive within the environment and host.

Results

Histoplasma morphology correlates with gain and loss of chromosome 7

Morphological shifts are critical to the pathogenic lifecycle of *Histoplasma*. As such, morphological bias—i.e., the phenotypic trait of favoring a particular morphology—is of great potential natural importance. We found that some *H. ohiense* strains were yeast-biased, continuing to grow as yeast for weeks in hyphae-inducing conditions, while other strains were more hyphal-biased, converting rapidly from yeast to hyphal growth when transitioned to ambient temperature. This morphological bias could be clearly observed by microscopy of cells grown in liquid culture (Fig 1A) as well as colony morphology (Fig 1B). Previously, a similar yeast-biased phenotype in liquid culture was mistakenly attributed to mutation of the *MSB2* gene, a finding that we could not replicate [22,23]. We thus subjected yeast- and hyphal-biased strains to whole genome sequencing.

Based on 6 strains with a partial second copy of chromosome 7 (Chr7), we found that hyphal bias correlated with a 334 kb region of Chr7, which we named the critical region (Fig 1C). Yeast-biased strains universally lacked an extra copy of this region. Hyphal biased isolates universally contained a second copy of this region.

We observed that yeast-biased isolates could spontaneously give rise to hyphal-biased isolates, and vice versa. Sixty-four strains, each generated concurrently with matched sibling strain(s) of the opposite morphology bias, were derived in multiple ways and from multiple genetic backgrounds (S1 Fig). To ensure a robust assessment of the correlation between morphology and CNV, some strains were selected randomly and subsequently assessed for morphology bias while other strains were selected based on morphology bias. Strains were then subjected to whole-genome sequencing. Of 26 hyphal-biased strains, 23 had duplication of the full Chr7, and all 26 showed duplication of the critical region. Of 38 yeast-biased strains, 36 were fully euploid, and all 38 lacked duplication of the critical region (Fig 1D).

Chromosome 7 aneuploidy is present in multiple *Histoplasma* isolates and species

Given the abundance of observed instances of Chr7 aneuploidy and the perfect correlation between CNV and morphological bias observed in the laboratory, we hypothesized that this aneuploidy would be relevant in natural populations. From analysis of previously published genome sequences [25–27], we found duplication of the full chromosome or the critical region in 16% of *Histoplasma* natural isolates of multiple species (Figs 2A and S2A–S2C). These data included CNV in the 6th largest chromosome of *H. mississippiense*, which is syntenic to *H. ohiense* Chr7 [24]. Individual natural isolates were subjected to short-term culturing, and then progeny were assessed for morphology bias and aneuploidy. Progeny displayed rough or smooth colony morphology, generally based on presence or absence of a hyphal ring indicating hyphal bias (Fig 2B). Sequencing of these isolates revealed that for 14/16 sequenced strains, colony morphology bias correlated with the CNV of interest, for both *H. mississipiense* and *H. ohiense* (Fig 2C and 2D). The remaining two sequenced strains displayed a unique phenotype of increased colony size and a rough variegated colony surface appearance rather than a rough fuzzy hyphal ring around the colony (4-Rough, Fig 2B). These two strains had a small CNV on chromosome 5 (Fig 2D). While CNV was observed among natural isolates in regions other than the aneuploid chromosome, CNV was by far most common in the aneuploid chromosome and was especially common in *H. ohiense* in the critical region (S2A–S2C Fig).

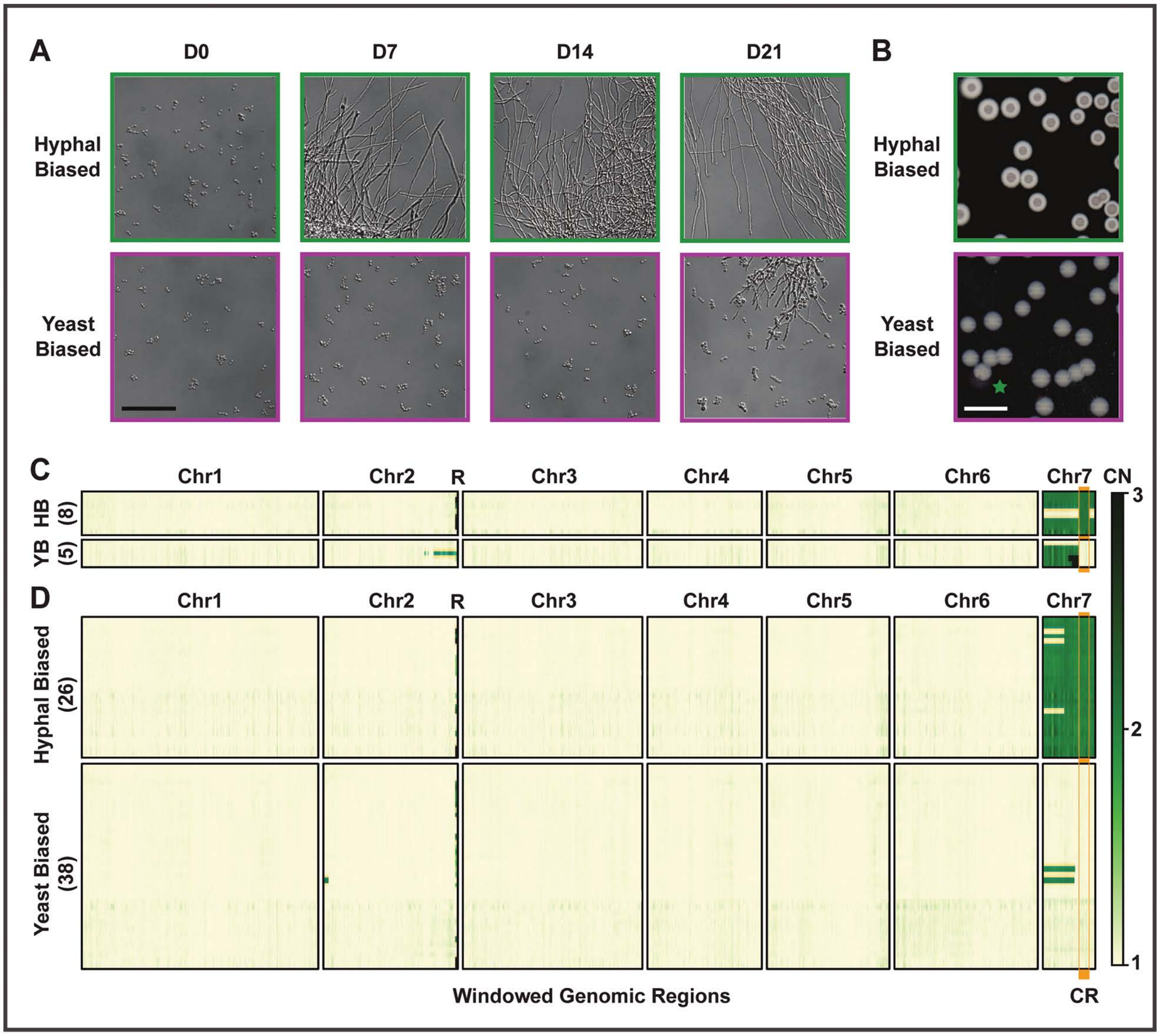

Fig 1. *Histoplasma* morphology correlates with aneuploidy of chromosome 7. Morphological bias of *Histoplasma* strains can be observed both microscopically as cell morphology and macroscopically as colony morphology. **A)** Representative microscopy images from strains with each morphology bias over a 21-day shift from 37 to 25°C. Bar indicates 50 μm. **B)** Representative images of colony morphology after 12 days at 25°C, including one yeast-biased colony with a potential sector of hyphal bias (green star). White bar indicates 10 mm. Green and magenta are used in A and B to indicate morphology bias as well as throughout this study. **C, D)** Windowed copy number (CN) based on normalized sequencing coverage of strains, excluding LTR retrotransposon blocks. Strains were categorized as hyphal-biased (HB) or yeast-biased (YB) prior to Illumina sequencing. The number of strains in each category is listed in parentheses on the *y* axis. A vertical orange bar is used to indicate the critical region ("CR"), the region of Chr7 that best correlates with morphology bias. The letter R indicates the location of ribosomal DNA on Chr2. **C)** Strains used to define the critical region and control strains. These include 6 strains with a partial second copy of Chr7. Closely related control strains with a full single copy or full double copy of Chr7 are also shown. **D)** Strains generated alongside one or more sibling strain of the opposite morphology bias. Correspondence between morphology bias and CR copy number is greater than expected by chance, as is correspondence between morphology bias and Chr7 copy number among strains with full 1× or full 2× Chr7 coverage. Fisher exact *p*-value $<1\times10^{-7}$ for both comparisons. All strains used here are derived from *H. ohiense* clinical isolate G217B. Additional information about the sources of strains in C and D is shown in S1 Fig.

https://doi.org/10.1371/journal.pbio.3003224.g001

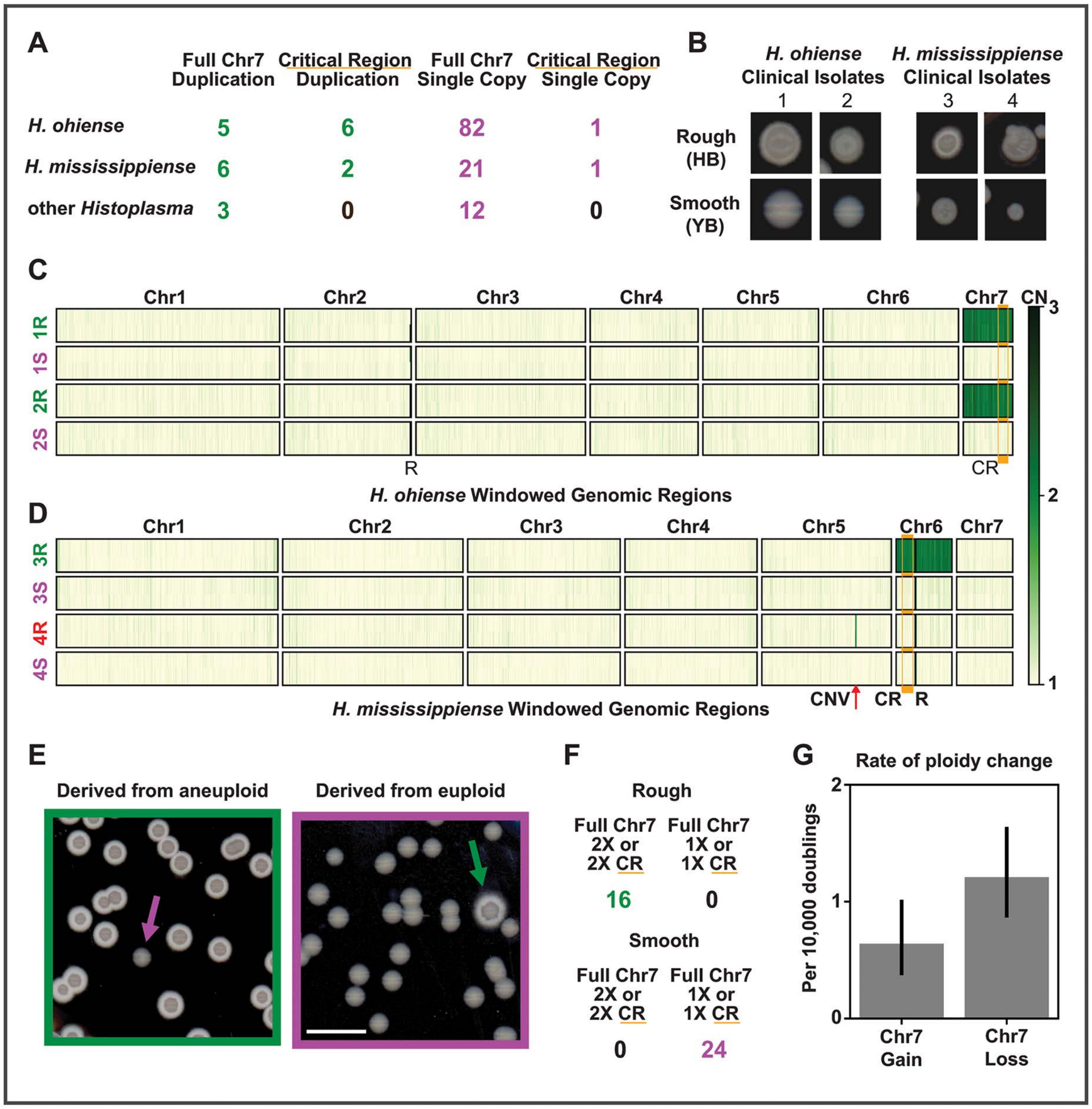

Fig 2. Ploidy variation correlating with morphology occurs in multiple *Histoplasma* species and is rapidly gained and lost. A) The Chr7 aneuploidy is present in natural population isolates of *H. ohiense*, *H. mississippiense*, and other *Histoplasma* sequenced by multiple previous groups. Columns from left to right indicate the number of isolates with a full second copy of Chr7, a second copy of a region of Chr7 including the critical region, only a single copy of Chr7, and a second copy of a region of Chr7 not including the critical region. **B)** Distinct colony morphologies were identified among progeny of two *H. ohiense* natural isolates (#1: CI_4, #2: CI_9) and two *H. mississippiense* natural isolates (#3: CI_43, #4: UCLA). For each of these four parental groups, morphology bias was categorized as smooth or yeast-biased (YB, e.g., category 1S from isolate 1) and rough or hyphal-biased (HB, e.g., category 1R). Clinical isolate 1 was imaged and categorized after 12 days at 25°C; isolates 2–4 were imaged and categorized after 10 days at 25°C. Progeny were subsequently sequenced, and copy number through the genome of these isolates is shown for *H. ohiense* strains **(C)** and *H. mississippiense* strains **(D)**. Strain categorization names are colored based on ploidy of strains in this category, with strains having a small CNV on chromosome 5 noted in red. The location of this small CNV is noted by "CNV" and a red arrow. As in Fig 1, critical region (CR) is noted by orange bar and rDNA is noted by the letter R. **E)** Example images of colonies from gain-and-loss rate experiments after 12 days at 25°C. Cells derived from an aneuploid parent (left, green) and colonies derived from a euploid parent (right, magenta) each showing one progeny colony (indicated by an arrow) that converted in morphology bias. Bar indicates 10 mm. Conversion in morphology bias was used as a proxy for converting in ploidy. **F)** 40 colonies from the assays used to determine gain and loss rates were sequenced to confirm morphology-CNV correlation. This sequencing is also included in Figs 1 and S1. **G)** Rates of Chr7 gain and loss determined by cell doublings and rate of morphology bias switching. Error bars show 95% confidence interval. The data underlying this Figure can be found in S9 Table.

https://doi.org/10.1371/journal.pbio.3003224.g002

The chromosome 7 aneuploidy is rapidly gained and lost

To assess the rate of gain and loss of the Chr7 aneuploidy, aneuploid and euploid yeast parental cells were subjected to short-term culturing at 37°C and ploidy of progeny was assessed. Importantly for this analysis, aneuploid and euploid yeast had indistinguishable growth rates (S2D Fig). We used colony morphology as a proxy for Chr7 aneuploidy and validated this assumption by sequencing 40 progeny colonies and obtaining a perfect correlation between ploidy of the critical region and morphology bias (Figs 2E, 2F, and S2E). We found that the rates of gain and loss of the Chr7 aneuploidy were 5.0×10^{-5} and 1.1×10^{-4} per doubling, respectively (Fig 2G). Surprisingly, these rates are significantly higher than the estimated rate of chromosome gain in haploid *Saccharomyces* and are comparable to the rate at which diploid *Saccharomyces* develops any individual aneuploidy [28–30].

Chromosome 7 aneuploidy confers a competitive advantage during the yeast-to-hyphal shift and a disadvantage during the hyphal-to-yeast shift

Given the high prevalence of the Chr7 aneuploidy and the high rate of gain and loss, we went on to assess if there might be conditions in which the aneuploidy confers a competitive advantage in a mixed population. We co-cultured an equal number of euploid and aneuploid cells and determined relative abundance of each population during temperature shift. A morphology score was assigned to each time point (S3 Fig). We found that in pooled competitions, the hyphal-biased aneuploid strains took over the population during the shift from yeast to hyphae at the timepoints at which hyphae were observed microscopically in these mixed ploidy cultures (Fig 3A–3C). Conversely, it was the yeast-biased euploid strains that took over the population during the reverse shift from hyphae to yeast (Fig 3D–3F). In both cases, this increased abundance could be due primarily to the morphology bias of each strain.

We also determined that significant Chr7 gain or loss within the population did not explain the observed changes in Chr7 copy number in these experiments. We used two spontaneously occurring non-coding SNPs as "barcodes" to track the starting strains. Euploid and aneuploid counterparts were generated for strains differing only at these barcodes and the appropriate pairs were mixed and subjected to morphology analysis and sequencing. Change in barcode ratios allowed us to determine that changes in the relative ratio of euploid to aneuploid cells was due to outcompetition rather than widespread Chr7 gain or loss over the course of the experiment (Fig 3A, 3B, 3D, and 3E).

In addition to mixed cultures, we also examined pure aneuploid and pure euploid cells under both transitions. In general, these cells maintained their ploidy state throughout the morphological transitions. However, we observed minor conversion of aneuploid to euploid cells in the hyphae-to-yeast transition as observed by the decrease in Chr7 copy number in the aneuploid population (Fig 3D). Conversion in this population was likely magnified by competitive advantage for euploid cells during the transition to yeast-phase growth. Comparing the timing of morphological transitions of pure populations and the mixed population also showed that the presence of aneuploid cells affects the speed of the transition of euploid yeast to hyphae: at day 7, the mixed population was almost fully hyphal despite the presence of many euploid cells while at this time point the pure euploid population was almost fully yeast.

Chromosome 7 aneuploidy reduces virulence and proliferation in a murine model of infection

To uncover how Chr7 gain or loss affects virulence, we used the murine model of infection to compare the virulence of aneuploid yeast, euploid yeast, and a 1:1 mix of the two. We found that cells with an extra copy of Chr7 were drastically reduced in their ability to cause lethal infection compared to their euploid counterparts (Fig 4A). Similarly, infections with aneuploid as opposed to euploid *Histoplasma* resulted in a significantly lower fungal burden by day seven both in the lung and spleen (Fig 4B). When a 1:1 mix of aneuploid and euploid *Histoplasma* was used for infection, mouse survival was similar to that of mice infected with a pure euploid population (Fig 4A). Interestingly, as the infection progressed, the percent of euploid cells significantly increased, a trend not found for yeast growing in vitro (Figs 4C and S4).

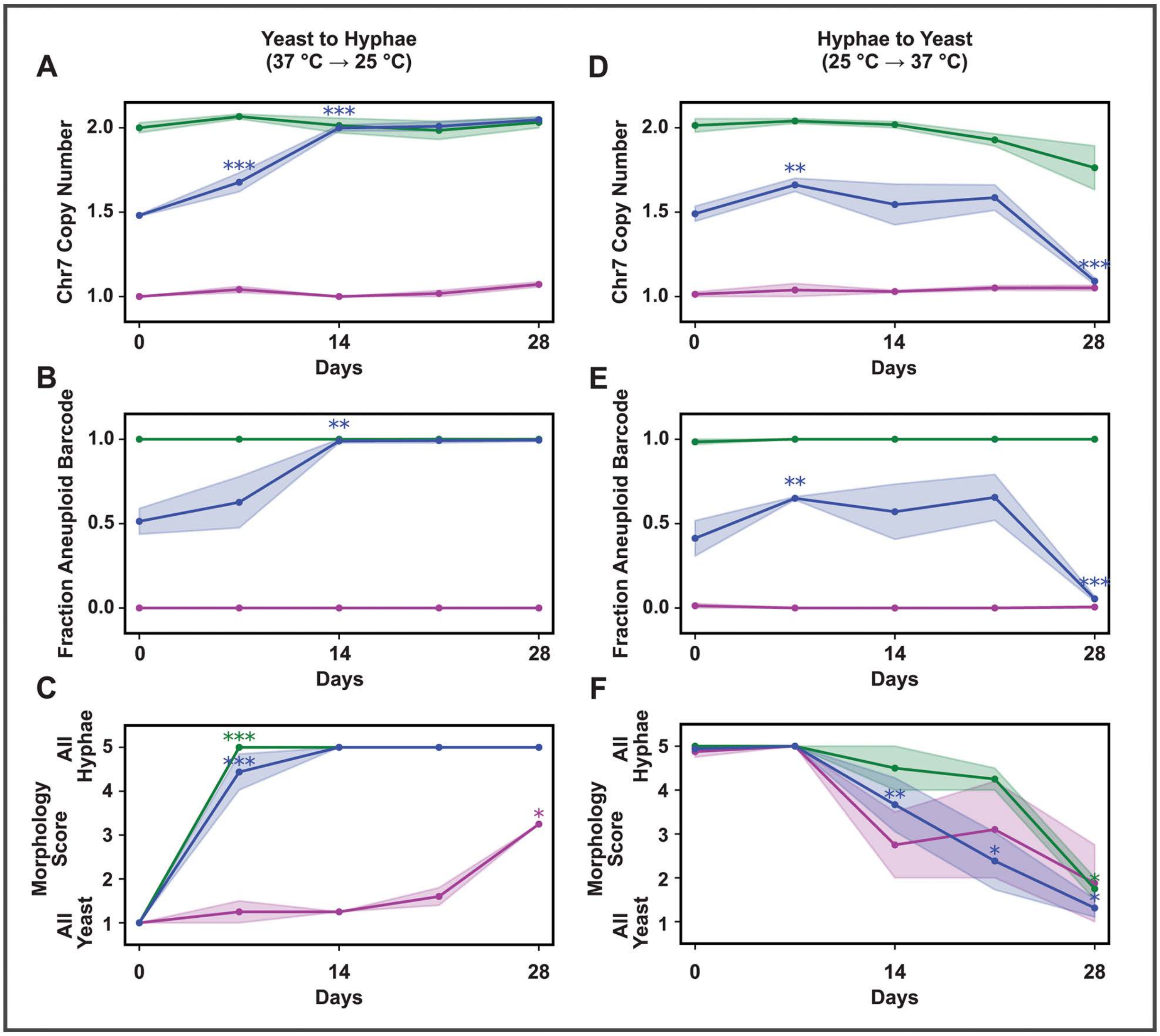

Fig 3. Chr7 aneuploidy confers a competitive advantage during the shift to the 25°C but confers a disadvantage during the shift to 37°C. Over the course of four weeks, yeast was transitioned to hyphae (moved on day 0 from yeast inducing conditions at 37°C with added CO_2 to hyphal inducing conditions at 25°C without added CO_2) and hyphae were transitioned to yeast (moved on day 0 from hyphae inducing conditions at 25°C without added CO_2 to yeast inducing conditions at 37°C with added CO_2). Transitions were performed separately for three starting populations: 50%−50% mix of aneuploid and euploid cells (blue), pure aneuploid (green), and pure euploid (magenta). **A–C** show the yeast-to-hyphae transition while **D–F** show the hyphae-to-yeast transition. **A, D)** Chr7 copy number throughout morphological transitions based on normalized sequencing coverage. **B, E)** Population ratios for each transition as fraction of aneuploid starting strain from SNP-based barcoding throughout transitions. **C, F)** Morphology, assessed by microscopy followed by image categorization, throughout morphological transitions. Morphology score ranged from 1 to 5 corresponding to all yeast (1), vast majority yeast (2), mixed (3), vast majority hyphae (4), all hyphae (5). We note as a caveat that cultures throughout the later portion of the hyphae-to-yeast transition exhibited notable flocculation which may increase morphology score variability. For each subfigure, asterisks show significance by *t* test of change vs. the prior point, colored based on population. The data underlying this Figure can be found in S10 Table.

https://doi.org/10.1371/journal.pbio.3003224.g003

Yeast with a second copy of Chr7 display a hyphal-biased transcriptome

We next assessed the *Histoplasma* transcriptome for ploidy-dependent trends with an eye towards mechanisms by which the aneuploidy might affect morphology bias or virulence. We performed RNA sequencing of aneuploid or euploid yeast,

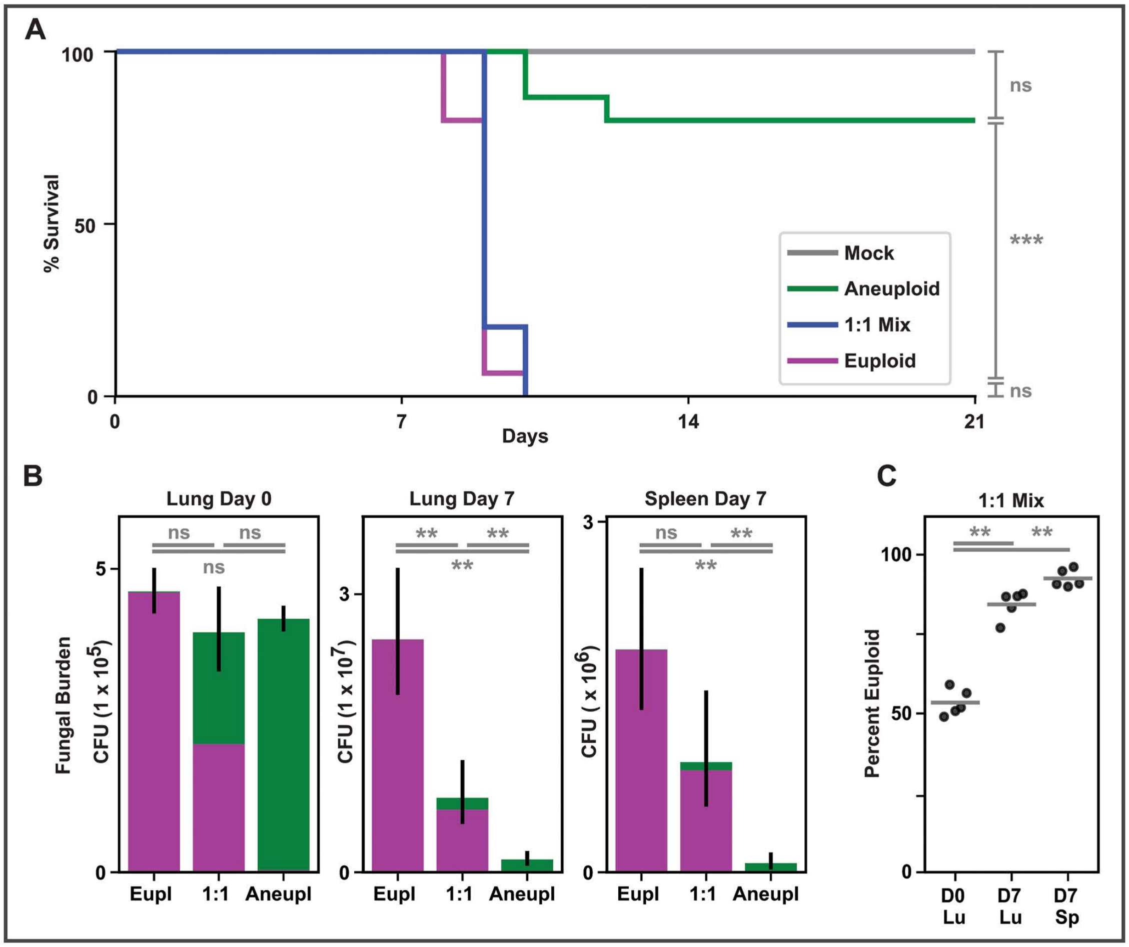

Fig 4. Aneuploidy reduces virulence in a mouse model. A) Survival curve of mice infected with aneuploid *Histoplasma* (green), euploid *Histoplasma* (magenta), a 1:1 mix of these two strains (blue), or mock infection (PBS, gray). Asterisks show significance based on log-rank test. **B)** Fungal burden by colony-forming unit (CFU) in organs of mice infected with *Histoplasma*, with colony morphology assay used to determine ploidy of fungi obtained from organs. Portion of CFU encompassing each ploidy is indicated by color in bar graph, with aneuploid shown in green and euploid shown in magenta. Asterisks show significance by Wilcoxon test of total CFU between infection categories. **C)** Percent of fungal burden encompassing smooth (euploid) colonies for each replicate with mean shown by line. Asterisks show significance by Wilcoxon test during 1:1 mix competition of portion of colonies that are euploid at Day 0 (hour 4 post-infection) in the lung vs. portion that are euploid in lung (Lu) and in spleen (Sp) at Day 7. The data underlying this Figure can be found in S11 Table (panel A) and S12 Table (panels B and C).

https://doi.org/10.1371/journal.pbio.3003224.g004

aneuploid or euploid hyphae, and aneuploid or euploid cells at two and seven days into the transitions from yeast to hyphae or hyphae to yeast (S5 Fig).

By comparing steady-state hyphae and yeast, we observed that the global transcriptional signature of hyphae in comparison to yeast correlated well between aneuploid and euploid cells (Fig 5A). Genes on Chr7 (shown in blue throughout Fig 5) displayed similar distribution of expression in hyphae over yeast as the remainder of the genome (Fig 5B). However, as expected, Chr7 transcripts were more abundant in aneuploid versus euploid cells (Fig 5C and 5D), concordant with increased copy number of those genes. We compared the expression profile of aneuploid and euploid yeast, as well as aneuploid and euploid hyphae. Out of the 11,795 total transcripts resolved in this experiment, ploidy affected twice as many transcripts in yeast (1,680 up and 1,667 down in aneuploid/euploid) than in hyphae (729 up and 800 down). Due to low correlation between the effects of ploidy on the two morphologies (Fig 5C) and a large number of ploidy-independent,

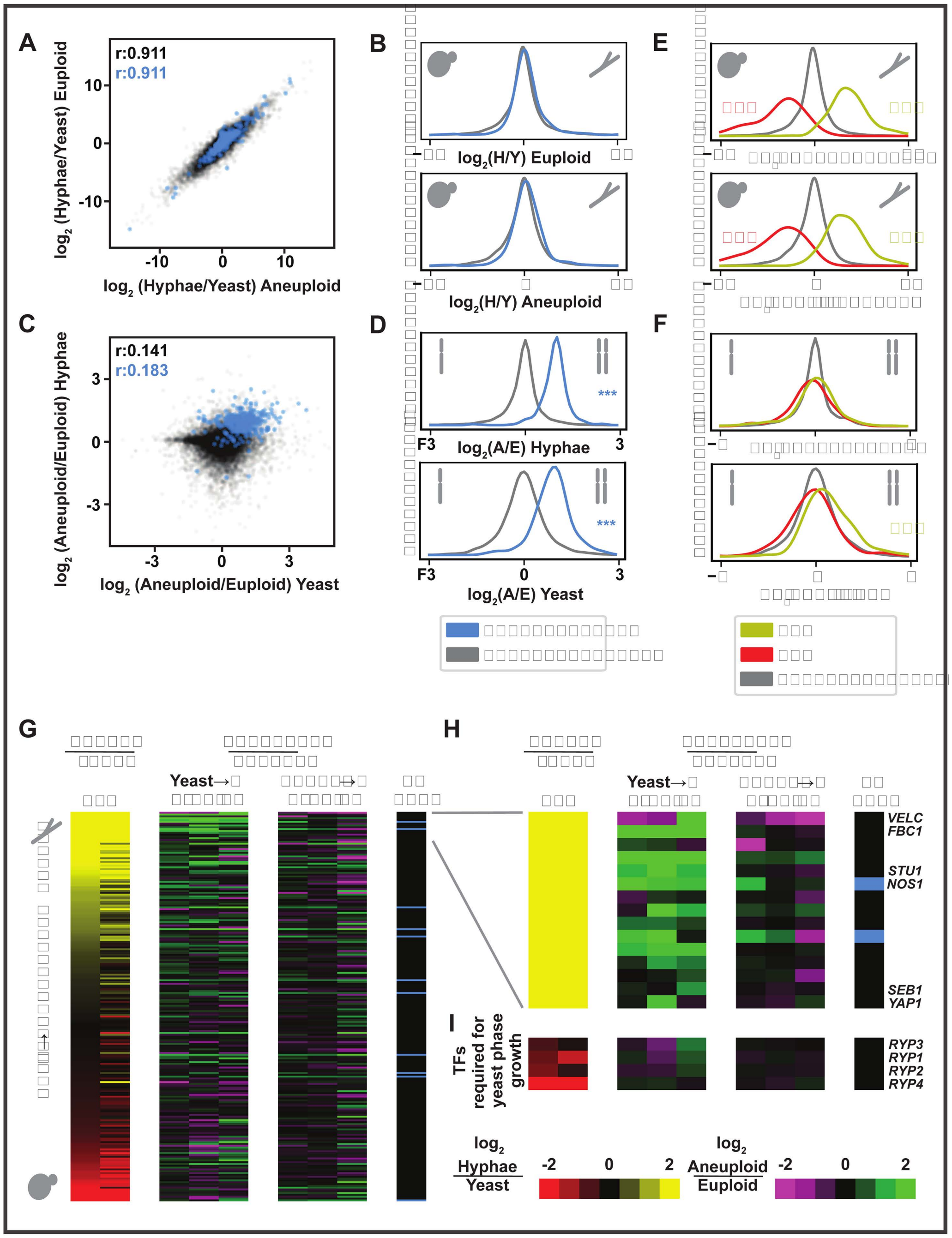

Fig 5. Aneuploid *Histoplasma* has a hyphal-biased transcriptome. A and C) scatter plots showing $\log_2$ ratios of transcript abundance. **A)** steady-state hyphae/steady-state yeast transcript ratios in aneuploid cells and in euploid cells. **C)** Aneuploid/Euploid transcript ratios in steady-state yeast and steady-state hyphae. Genes on chromosome 7 are shown in blue, all other transcripts are shown in gray. Pearson's r correlation coefficients are shown in the upper left, note different axis ranges in A and C. **B, D–F)** Distribution of $\log_2$ ratios of transcript abundance, showing the same set of axes as used in A and C. Ratios from top to bottom include euploid hyphae/euploid yeast, aneuploid hyphae/aneuploid yeast, aneuploid hyphae/euploid hyphae, aneuploid yeast/euploid yeast. **B, D)** Transcripts encoded by genes on Chr7 are highlighted in blue. **E, F)** YPS transcripts are shown in red and HPS transcripts are shown in yellow. Asterisks are shown if significant by Wilcoxon test and if distribution median is least 5% from center to plot maximum or minimum. **G)** Heatmap showing all predicted transcription factors. The first two columns show transcript abundance in hyphae/yeast (column 1: euploid, column 2: aneuploid) (yellow vs. red). Subsequent columns show transcript abundance in aneuploid/euploid cells through yeast-to-hyphal transition (37–25°C) and hyphal to yeast transition (25–37°C) (green vs. magenta). For each time point of each transition (steady state, day 2, and day 7), abundance

for each plotted transcript is derived from 3 replicates. Final column indicates transcripts encoded by genes on Chr7 in blue. **H)** Expanded view of the top 15 genes in G with named genes labeled. **I)** The same set of columns is shown for transcription factors required for yeast phase growth (*RYP1-4*). The data underlying this Figure can be found in S3 Table.

https://doi.org/10.1371/journal.pbio.3003224.g005

temperature-dependent transcripts (S5F Fig), the overall yeast- and hyphal-enriched programs remained correlated in the two genetic backgrounds, as noted above.

We also analyzed differential expression for sets of previously defined *Histoplasma*-conserved yeast-phase specific transcripts (YPS) and hyphal-phase specific transcripts (HPS) [20]. We found that YPS had the expected increased abundance in yeast versus hyphae independent of ploidy just as HPS had the expected increased abundance in hyphae versus yeast (Fig 5E). We found that HPS transcripts were also significantly more abundant in aneuploid versus euploid yeast (Fig 5F). These data indicate that aneuploid yeast show a pre-existing hyphal bias in their transcriptome, likely underlying the ability of these yeast to more rapidly undergo conversion to hyphae upon temperature shift.

We also assessed differential expression of TFs in this experiment based on a previously defined list of putative TFs [31]. We found that the TF transcripts that are most abundant in hyphae relative to yeast were often also more abundant in aneuploid yeast relative to euploid yeast, consistent with the hyphal bias of these cells (Fig 5G and 5H). These TF transcripts showed even higher differential expression in aneuploid versus euploid cells during the transition from yeast to hyphae compared to steady-state yeast. Once aneuploid and euploid cells transitioned to steady-state hyphae, their transcriptomes looked more similar and TF transcript abundance was no longer as differential. Similar trends were not observed in the hyphal to yeast transition (Figs 5G and S5C), consistent with ploidy having a larger effect on yeast than on hyphae.

Two of the TFs that were upregulated in aneuploid cells, *STU1* and *FBC1*, neither of which are on Chr7, have been shown to be sufficient to induce the hyphal morphology when ectopically expressed in cells at 37°C [21]. However, we found that transcript abundance of the TFs required for yeast phase growth (*RYP1–4* [17,19]) were not strongly regulated by ploidy (Fig 5I). Taken together, these data suggest that the transcriptome of aneuploid yeast renders these cells more primed for the hyphal transition compared to euploid yeast.

Although the YPS regulon is large, only a handful of genes have been tested for virulence in the mouse model. Five *Histoplasma* genes have been shown to affect mouse survival during infection [32–35], none of which are significantly regulated in aneuploid versus euploid yeast (S5D Fig). However, among a recently defined set of 11 additional predicted virulence effectors [35], 5 are significantly more expressed (mean 4.5 fold increase) in euploid versus aneuploid yeast (S5E Fig).

Identification of a transcription factor on chromosome 7 that promotes hyphal bias

Our analysis of matched yeast-biased and hyphal-biased strains allowed us to narrow down the region that was necessary for hyphal bias to a 334 kb critical region on Chr7 (Fig 1C). This critical region contains 145 predicted genes (1.2% of genes in the genome), including some with minimal evidence of expression and no predicted protein domains. None have been studied previously in *Histoplasma*. Through manual curation of the genes within this region (considering Pfam domain annotations, *Histoplasma* expression patterns, and annotated homologs), we generated five regions of interest, some containing >1 gene. The corresponding genes of interest included the only predicted TF in the region, two genes with distant homologues that affect morphology (protein kinase (PK) *YAK1* and chromatin modifying component *ADA2* [36–38]), other PKs, and one gene encoding a small hyphal peptide (SHP) of unknown function with strongly hyphal-enriched transcription and translation [20]. Each of these five subregions was cloned into individual plasmids that were transformed into aneuploid and euploid *Histoplasma* strains (Figs 6A and S6A–S6E) and the resultant effect on hyphal bias was determined.

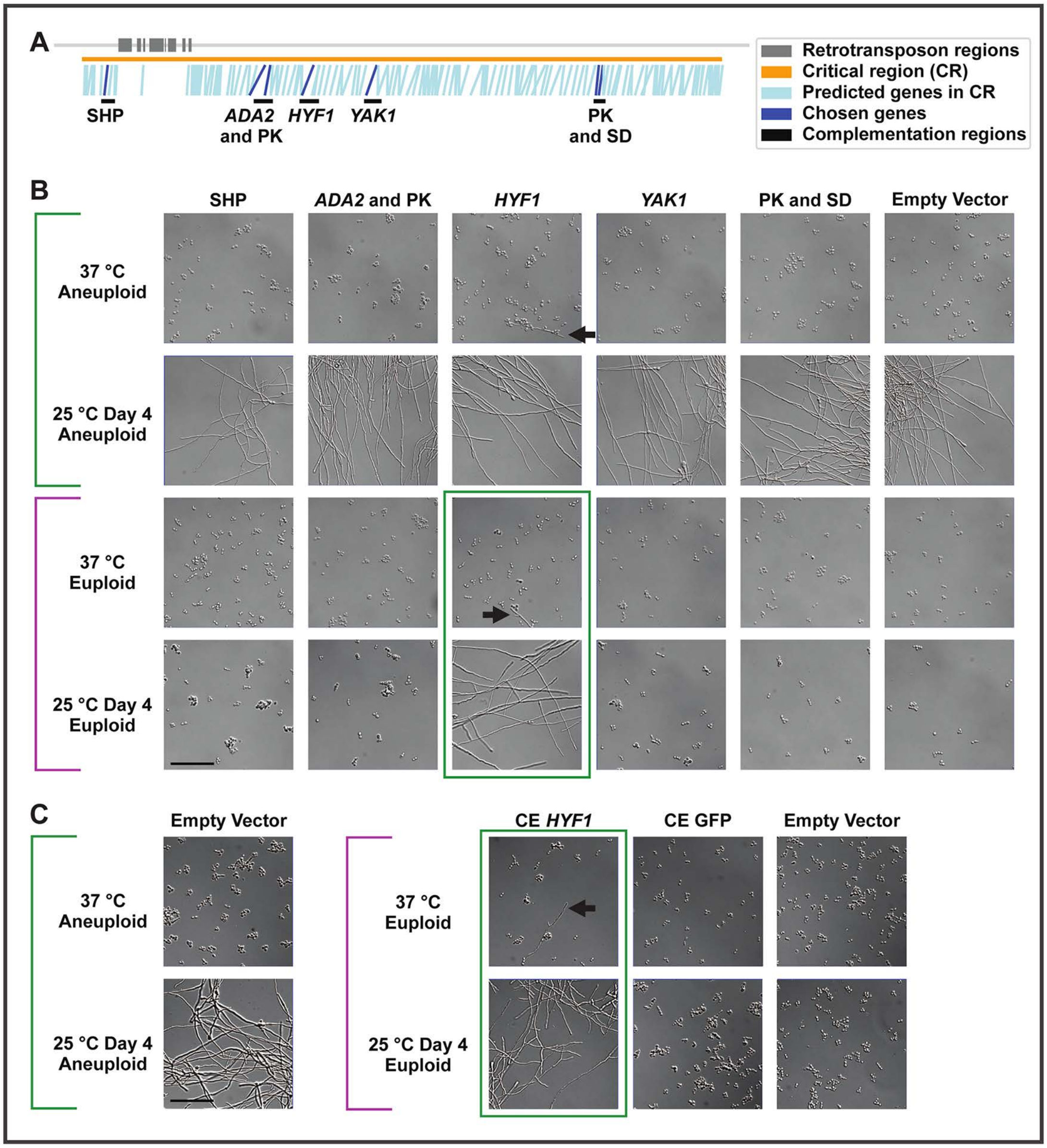

Fig 6. A chromosome 7 transcription factor, *HYF1*, is sufficient to confer hyphal bias. A) Locations of genes (blue) in the critical region (orange) with retrotransposon regions (dark gray) and regions chosen for increased copy number (black). Primary gene(s) chosen for each region are labeled and shown in dark blue. The critical region is 334 kb and contains 145 predicted genes. Chosen regions from left to right contain a small hyphal peptide (SHP), the chromatin modifying component *ADA2* (region also includes a PK), the TF *HYF1*, the PK *YAK1*, a region with a PK and a superoxide dismutase (SD). **B)** Microscopy of aneuploid and euploid cells at 37°C or after 4 days at 25°C with ectopic plasmids containing a portion of the critical region (to increase copy number) or an empty vector. **C)** Microscopy of aneuploid and euploid cells at 37°C or after 4 days at 25°C with constitutive expression (“CE”) (using the *ACT1* promoter) of *HYF1* or GFP (control) or an empty vector. In B and C, scale bar indicates 50 μm. Brackets and boxes in magenta and green indicate morphology bias. Black arrows indicate hyphal filaments in images dominated by yeast.

https://doi.org/10.1371/journal.pbio.3003224.g006

Although four gene segments had no effect on hyphal bias, increasing the copy number of the critical region TF on a plasmid under the control of its native regulatory elements was sufficient to confer hyphal bias in a euploid background (Fig 6B). In fact, a very small number of hyphal cells were visible in these strains even at 37°C, unlike control aneuploid

or euploid strains that contained no hyphae. This may be related to the fact that average copy number of this plasmid in yeast was above 1×, at 1.4× based on normalized bulk sequencing coverage at 37°C. We named this TF *Hyphae Promoting Factor* (*HYF*) *1*. *HYF1* is orthologous to *CON7,* which affects morphology and virulence phenotypes in multiple fungal species [39–41], and orthologous to *WOR4,* which drives the white-to-opaque transition in *Candida albicans* [42] (S6F Fig). Unfortunately, we were not able to successfully use CRISPRi to generate knockdown strains of *HYF1* despite several attempts. Constitutive expression of *HYF1* under the control of the *ACT1* promoter in euploid cells triggered strong hyphal bias, suggesting that this TF is a key regulator of hyphal development (Fig 6C).

Increased copy number of *HYF1* recapitulates the transcriptome hyphal bias associated with increased copy number of Chr7

To determine if increased copy number of *HYF1* was sufficient to shift the transcriptome in the same manner as increased Chr7 copy number, we subjected euploid yeast carrying a plasmid encoding *HYF1* with its native regulatory elements to transcriptional profiling. The effects of this plasmid on transcript abundance were compared to those observed in aneuploid versus euploid yeast. As expected, Chr7 CNV significantly affected the abundance of many more transcripts than increased *HYF1* copy number alone (Fig 7A and 7B). Additionally, a higher portion of transcripts with increased abundance in aneuploid cells versus transcripts up in the *HYF1*-bearing strain are on Chr7 (30% versus 5%). 144 transcripts exhibited increased expression under conditions of both increased Chr7 copy number and increased *HYF1* expression. These transcripts were enriched for the previously defined HPS, which are highlighted in Fig 7C. In contrast, the effect on the bulk of YPS was minimal (Fig 7D).

Most TF transcripts with increased abundance in the aneuploid strain were either on Chr7 or instead also had increased abundance in response to increased *HYF1* copy number (Fig 7E). Of the 15 most abundant TFs in aneuploid versus euploid cells, 5 TFs were encoded on Chr7, and these TFs were not upregulated under conditions of *HYF1* increased copy number. In contrast, six of the remaining 10 TFs did show increased abundance under conditions of increased *HYF1* copy number (Fig 7F). The two most differentially abundant TFs among these six, *FBC1* and *STU1*, have been previously shown to be sufficient to induce hyphal growth [21]. Increased expression of *STU1* and *FBC1* may be part of the mechanism by which increased copy number of *HYF1* induces hyphal bias.

It is possible that, while not required for hyphal bias, additional genes on Chr7 may also contribute hyphal phenotypes associated with aneuploidy. We also assessed the effects of increased copy number of an additional TF on this Chr7 found directly adjacent to the critical region that is orthologous to *RFEC (FFMA)* which is required for normal hyphal growth in *Aspergillus* [43]. We named this TF *HYF2*, expressed it on a plasmid in euploid cells, and found that it recapitulated some of the morphological and transcriptomic phenotypes of cells carrying a second copy of Chr7 (S7 Fig). However, its effect on both morphology and transcriptome was much more subtle than that of *HYF1*.

These data defined a *HYF1* regulon of transcripts whose abundance changed in response to increase in *HYF1* copy number. To determine if the *HYF1* regulon is broadly correlated with filamentous growth in other fungi, we assessed the morphology-specific expression of these genes in available fungal yeast versus hyphae datasets (Fig 7G) [20,31,44–50]. Within the thermally dimorphic pathogens in pezizomycotina, there was significant enrichment of *HYF1* regulon orthologs among genes up in hyphae. In contrast, we did not observe this enrichment for the close *Sporothrix* relative *Ophiostoma novo ulmi*, a plant pathogen with temperature-independent dimorphism. Likewise, the enrichment did not hold for the more distantly related ascomycete *C. albicans*, a mammalian pathogen with a 37°C-induced yeast to hyphal transition, nor for the basidiomycete plant pathogens *Meira miltonrushii* and *Tilletiopsis washingtonensis* which, like *Ophiostoma*, have temperature-independent dimorphism.

Notable genes with conserved hyphal expression in most or all of the thermally dimorphic fungi included the ortholog of *A. nidulans* developmental regulator *esdC* [51], the ortholog of *Metarhizium anisopliae* cold shock protein *CRP2* [52], and the TFs *FBC1* and *STU1*. The conserved hyphal expression for *STU1* in particular was striking. It was up at least 2-fold in

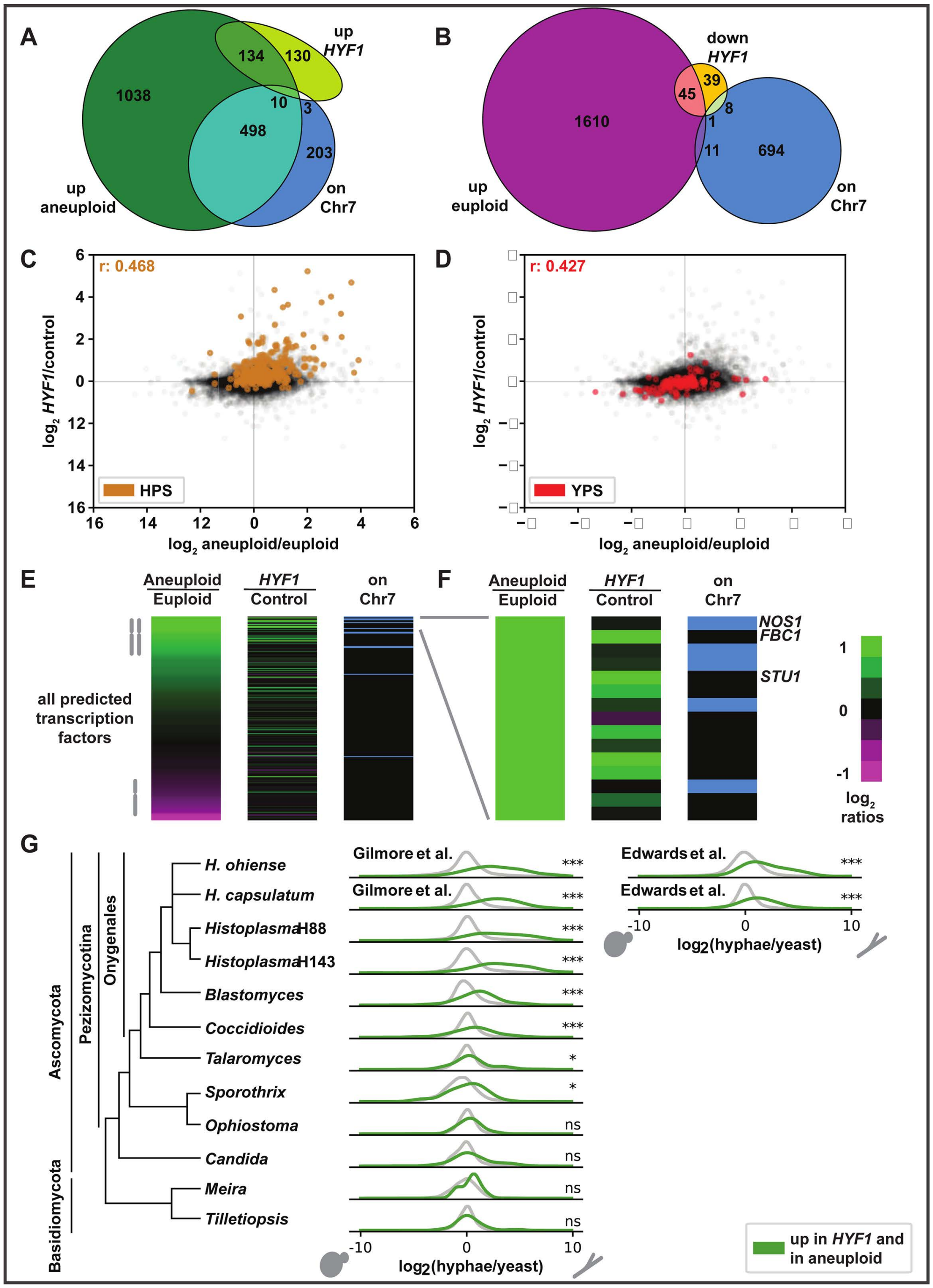

Fig 7. *HYF1* recapitulates some of the key transcriptional effects of aneuploidy. A) Venn diagram showing transcripts that reach significance and 1.5-fold increase in aneuploid vs. euploid yeast (green) and *HYF1* increased copy number over empty vector control yeast (lime) and genes on chromosome 7 (blue). **B)** Venn diagram showing transcripts that reach significance and 1.5-fold increase in euploid vs. aneuploid yeast (magenta), 1.5-fold decrease in *HYF1* increased copy number vs. control yeast (orange) and genes on chromosome 7 (blue). **C, D)** Scatter plots showing log_2 ratios of transcripts in aneuploid vs. euploid yeast (x-axis) and *HYF1* increased copy number over control yeast (y-axis) highlighting HPS genes (brown, in **C**) or YPS genes (red, in **D**). **E)** Heatmap showing all predicted TFs with the same two ratios as in C, D followed by a column indicating which genes are on Chr7 (blue). TFs are sorted based on the first column. **F)** The top 15 transcripts from E. **G)** Distribution of yeast vs. hyphae transcript abundance in orthologs of all *Histoplasma* genes (gray) and genes up in *HYF1* increased copy number and aneuploid (green). Asterisks indicate significance by Wilcoxon. The data underlying this Figure can be found in S4 Table (panels **C–F**) and S7 Table (panel **G**). Data sources for S7 Table are listed in S6 Table.

https://doi.org/10.1371/journal.pbio.3003224.g007

hyphae in all of the pezizomycotina, with only *Ophiostoma* failing to pass the 5% FDR significance criterion, and statistically significant but, at 1.99-fold, just under the fold-change criterion in *Meira*.

As most of the species with conserved hyphal expression of the *HYF1* regulon are both thermally dimorphic pathogens of mammals and also the most closely related to *Histoplasma*, we could not distinguish whether this expression pattern is a feature of this class of pathogens versus a conserved property of pezizomycotina versus a more general pattern that is obscured in more distant relatives of *Histoplasma* due to increased difficulty of ortholog mapping. Nevertheless, the stronger hyphal enrichment of the *HYF1* regulon in *Sporothrix* relative to *Ophiostoma* suggests that this enrichment may be a property of thermal dimorphs.

To further understand the role of *HYF1*, we examined the effect of constitutive expression of *HYF1* as yeast transition to hyphae by subjecting euploid yeast carrying P_{ACT1}-*HYF1* or an empty vector to transcriptional profiling at 37°C and after a two-day transition to 25°C. *HYF1* transcript levels were up just 1.2-fold when *HYF1* was expressed under the control of the *ACT1* promoter at 37°C, somewhat less than was observed in aneuploid/euploid yeast at 37°C (1.9-fold) or yeast carrying a *HYF1* plasmid with native regulatory elements/control (1.4-fold) (S8A Fig). Interestingly, *HYF1* transcript levels were not significantly differential in P_{ACT1}-*HYF1*/control or aneuploid/euploid after a two-day transition to 25°C. Despite equivalent *HYF1* transcript abundance after a two-day transition to 25°C, transcripts in the *HYF1* regulon continued to show higher abundance in P_{ACT1}-*HYF1*/control ($p = 6.2e^{-06}$, S8B Fig). This finding indicates that increased transcript abundance of *HYF1* was not necessary to sustain expression of the regulon during this transition. Rather, *HYF1* was sufficient to induce the regulon at 37°C ($p = 9.2e^{-14}$, S8C Fig) and prime cells for quicker transition to hyphae upon exposure to 25°C.

Discussion

We discover here that gain or loss of an extra copy of a particular *Histoplasma* chromosome, a reversible genetic change, has significant effects on the switch between the environmental and host forms as well as the ability of the organism to thrive during infection. In the laboratory, gain or loss of this chromosome occurs at the rate of $5-11 \times 10^{-5}$ per generation, and our detection of CNV of this chromosome in 16% of previously sequenced natural isolates indicates that it occurs broadly. Notably, euploid cells have a significant competitive advantage in the host whereas cells with an extra copy of this chromosome have an enhanced ability to switch to the environmental form in response to temperature cues.

Facile gain and loss of a specific *Histoplasma* chromosome may benefit *Histoplasma* by increasing phenotypic variability, helping populations survive abrupt transitions between environment and host (Fig 8). It may be beneficial for a subpopulation of cells to be primed to switch morphology while another subpopulation is recalcitrant to fluctuations in stimuli including temperature, which can overlap between human airways and the soil [53]. Hyphal cells are occasionally observed in human tissues in cases of severe histoplasmosis, and it is possible that this morphological diversity could be evolutionarily beneficial to *Histoplasma* in preparation for possible transition to environmental growth [54–56]. We hypothesize that Chr7 aneuploidy may act as a bet-hedging system, allowing variability in the morphological response. We also find it interesting that in mixed aneuploid–euploid cultures transitioning from yeast to hyphae, the presence of aneuploid cells seems to promote euploid cells to transition from yeast to hyphae more rapidly. It is possible that in addition to functioning as a bet-hedging system, successful hyphal growth of a subpopulation may act as a herald, inducing morphological transition in more of the population. Successful growth as hyphae implies a good environment for hyphae, therefore the initial hyphal cells might send a positive feedback signal, e.g., by production of a secreted factor that promotes hyphal growth, to induce the transition in more of the population. Indeed, there is precedent in other fungi for secreted factors that influence morphology via quorum-sensing mechanisms [57–59].

Since this CNV is common in sequenced natural isolates and prevalent in lab strains, it is intriguing to speculate that the aneuploidy could have influenced prior experimental findings relevant to *Histoplasma* virulence or filamentation,

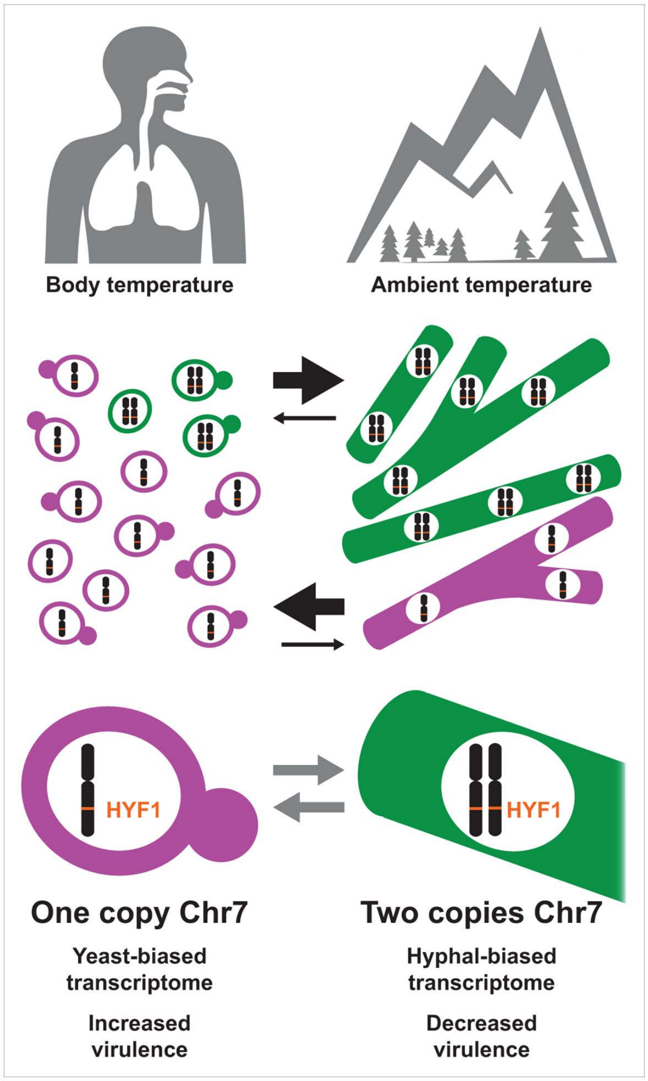

Fig 8. Rapid gain and loss of Chr7 aneuploidy may increase phenotypic diversity. Our data suggest that rapid gain and loss of the Chr7 aneuploidy may benefit *Histoplasma* by rapidly increasing phenotypic diversity, helping populations survive frequent and abrupt transitions between environment and host. *Histoplasma* grows as yeast in the mammalian body and in the laboratory when grown at 37°C, but as hyphae in the environment or in the laboratory when grown at 25°C. Cells with a second copy of chromosome 7 are biased towards hyphal growth and outcompete euploid cells in the yeast-to-hyphal transition (black arrows). Euploid cells (with one copy of each chromosome) are biased towards yeast growth and outcompete in the hyphal to yeast transition (black arrows). Cells frequently gain and lose a second copy of Chr7 (gray arrows). Cells with one copy of Chr7 have increased virulence in comparison to cells with two copies of the chromosome. Cells with two copies of Chr7 have a hyphal-biased transcriptome as do cells with increased copy number of *HYF1*, a TF on Chr7.

https://doi.org/10.1371/journal.pbio.3003224.g008

especially those involving genetic manipulation which involves passaging of strains. In contrast, the discovery that GlcNAc affects the speed of the transition to hyphae [60], is unlikely to be influenced by ploidy, and the effects of CNV were clear both with and without the addition of GlcNAc (e.g., Fig 1A without GlcNAc and Fig 6B with GlcNAc). At minimum, this CNV explains the phenotypes previously attributed to *MSB2* in *Histoplasma* which were identified through comparison of an aneuploid "wild-type" strain and a euploid *msb2* mutant [22,23]. While ploidy variation is clearly observed in human clinical isolates of *Histoplasma*, we do not yet know if *Histoplasma* ploidy variation correlates with clinical presentation or is

influenced by the immune status of the host. Although the mouse model of infection selected for euploid *Histoplasma*, this selection could be affected by clinical variance among humans such as inoculum dose, speed of disease progression, and variation in the immune response. Exceedingly few environmental isolates are available, so it remains to be seen the level at which ploidy variation is present in *Histoplasma* growing in the environment.

Our work revealed that aneuploidies in *Histoplasma* are by far most common on Chr7 versus other chromosomes. In contrast to *Histoplasma*, the incidence of aneuploidy is often spread more evenly between chromosomes in model species although certain aneuploidies are associated with adaptation to specific stressors [28,29,61–63]. While it is possible that relative rate of Chr7 aneuploid acquisition (e.g., increased nondisjunction of Chr7 versus other chromosomes) may contribute to the frequency at which we observe Chr7 CNV, we hypothesize that the primary driver is relative aneuploid fitness burden among the seven chromosomes. For both *H. ohiense* and *H. mississippiense*, the aneuploid chromosome contains the fewest genes, which could be related to a lower fitness cost [64]. However, partial chromosome CNV is also most common within Chr7. We thus hypothesize that minimal fitness cost of duplication of genes on Chr7 and conditional competitive advantage of this CNV are the primary drivers of its frequency.

Identification of the critical region facilitated our discovery of the TFs *HYF1* and *HYF2* as hyphae-promoting factors. While these genes have not been investigated previously in *Histoplasma*, each is orthologous to a gene with functions related to morphology and cell fate. *HYF1* is orthologous to the *C. albicans* TF *WOR4*, which is involved in a transcriptional network regulating white-opaque switching [42]. *Histoplasma* orthologs of other genes within this *Candida* network are also involved in the *Histoplasma* regulation of morphology, including *RYP1*, an ortholog of *Candida WOR1*, and *STU1*, an ortholog of *Candida EFG1* [17,42]. *HYF2* is orthologous to the *Aspergillus fumigatus* protein *FFMA*, whose deletion results in reduced growth [43]. Thus far, we have not been able to successfully generate knockdown strains targeting *HYF1* or *HYF2*. Genetic manipulation can be challenging in *Histoplasma*, so it is unclear if the lack of a knockdown strain indicates that, like *FFMA*, *HYF1*, and *HYF2* are required for normal growth. While a minority of cells transition to hyphal growth in response to an increase in *HYF1* copy number even at body temperature, for most cells, the increase in *HYF1* copy number potentiates the morphological response to lowered temperature. The Hyf1 transcript or protein, or a transcript or protein whose abundance is affected by *HYF1* such as Fbc1 or Stu1, may in some way be activated by a change in temperature to trigger this morphological response.

The effect of Chr7 CNV was more evident in yeast than in hyphae. Aneuploid yeast transitioned to hyphae much more quickly than euploid yeast transitioned to hyphae. However, aneuploid and euploid hyphae demonstrated only minor differences in speed of conversion to yeast. Similarly, while we observed a hyphal bias in the transcriptome of aneuploid yeast, suggesting that they were primed to transition more quickly to hyphae, euploid hyphae did not display as much yeast bias in their transcriptome. It is possible that some of the observed relative effect of ploidy may be attributable to our better understanding of the yeast-to-hyphal transition than our understanding of the hyphal-to-yeast transition. The transition to yeast cells can either occur directly from hyphae, or via generation of conidia from hyphae followed by germination of conidia to give rise to yeast cells. It is possible that aneuploidy might affect the rate of conidia production and/or germination into yeast, but given technical limitations in robust and reproducible conidiation, we did not measure the rates of these processes. The yeast-to-hyphal transition requires less time than the reverse transition and is more synchronous under current culture conditions (*c.f.* variance in Fig 3C versus Fig 3F), and is thus more commonly performed in the laboratory. In fact, this is the first publication of transcriptional profiling of hyphal cells shifted to yeast-inducing conditions, providing a dataset that will be relevant to subsequent investigations of thermal dimorphism.

Our observation that a reversible chromosomal aneuploidy has significant effects on morphologic transitions and disease is essential to understanding thermal dimorphism and pathogenesis in this important group of understudied human pathogens. Although epigenetic changes that drive switches are more commonly reported, specific reversible genetic changes akin to this *Histoplasma* aneuploidy have been observed previously [65–69]. Such changes include inversion of DNA sequences, homologous recombination between transposons, and aneuploidies, and likely often have an

intermediate stability between that of epigenetic modifications and standard genetic mutations. These reversible genetic changes often confer benefits in a subset of the ecological or pathogenic niches inhabited by a microbe. We posit that similar yet unidentified genetic changes are likely important to the pathogenesis of many additional microbes, conferring semi-transient diversity to improve population survival through repeatedly fluctuating conditions.

Methods

Ethics statement

All animal work was approved under UCSF Institutional Animal Care and Use Committee protocol AN197403.

Histoplasma growth conditions

Yeast were grown at 37°C with 5% CO_2, and hyphae were grown at 25°C without CO_2. Culture of hyphae was performed in a Biosafety Level 3 facility. For liquid growth, cells were grown in *Histoplasma*-macrophage medium (HMM) in an orbital shaker at 120–150 RPM. During liquid growth, cells were passaged at a 1:25 dilution every 2–3 days for yeast and weekly for hyphae. For growth on plates, HMM agarose was used. Plates and liquid HMM were supplemented with 0.2 mg/mL uracil for growth of uracil auxotroph strains. If indicated, the glucose in HMM media was replaced with equimolar N-acetyl-glucosamine (GlcNAc) to increase the speed of transition from yeast to hyphae [60].

Histoplasma strains

Strains used to assess morphology-CNV correlations are derived from clinical isolates G217B, CI_4, CI_9, CI_43, and UCLA as described for Figs 1 and 2. The *URA5* deletion strain WU15 [72], which was derived from *H. ohiense* clinical isolate G217B is otherwise used unless noted. This includes otherwise isogenic pairs of aneuploid and euploid strains used for experiments shown in Figs 1A, 1B, 2E–2G, 3, and 5. WU15 + *URA5* is used for mouse infections shown in Fig 4. One non-genic SNP was present between euploid and aneuploid strains used in this experiment. Otherwise isogenic pairs of aneuploid and euploid WU-15 strains were transformed with plasmids to obtain strains used in Figs 6 and 7 as described below. Strains used in this paper are also described in S1 Table.

Assessment of morphology and morphology bias categorization from colony appearance and microscopy

To generate single colonies to assess colony appearance, cells in a yeast culture were briefly sonicated, counted with a hemocytometer, then plated to obtain around 40–100 colonies per plate. Plates were grown at 37°C and 5% CO_2 until colonies were small but clearly visible, around one week, then moved to 25°C without CO_2. Colonies were allowed to grow until the morphology differences between yeast-biased and hyphal-biased controls were stark, around 10 additional days. HMM plates were not supplemented with GlcNAc for this analysis.

For categorization of morphology bias by microscopy of liquid cultures, yeast cultures were passaged 1:25 into HMM with GlcNAc on day −2 and then on day 0 were passaged to OD_{600} 0.2 in HMM with GlcNAc and then moved from 37 to 25°C. This protocol was also used in transitions to assess morphology bias of strains with ectopic plasmids (e.g., Fig 6). Microscopy samples were taken at subsequent days as indicated for individual experiments and images. Samples for morphology bias categorization were taken between days 2 and 10 when sharply delineated morphology differences were consistently observable for control strains. Yeast-biased isolates had morphology scores of 1 (all yeast) or 2 (vast majority yeast) while hyphal-biased isolates had morphology scores of 4 (vast majority hyphae) or 5 (all hyphae). A representative yeast-biased euploid isolate and an otherwise isogenic hyphal-biased aneuploid isolate were transitioned again with the same protocol but without GlcNAc for the microscopy images shown in Fig 1A. For imaging, cells were fixed in 4% PFA for 30 min and stored at 4°C until DIC imaging on a Zeiss AxioCam MRM microscope.

DNA sequencing and analysis, including ploidy categorization

Genomic DNA extractions and sequencing were performed as previously described [24]. All DNA extractions other than for the five dual fluorescent strains were performed using the bead beating protocol [24]. Genomic DNA extraction for the five dual fluorescent strains were performed using the slightly modified protocol based on the Qiagen Gentra Puregene Yeast/Bacteria kit (158567) [24]. Illumina sequencing was performed at UCSF Center for Advanced Technology, the Chan Zuckerberg Biohub—San Francisco, or SeqCenter, LLC (Pittsburgh, PA). Reads were aligned to assembly UCSF3 for non *H. mississippiense* strains and to the WU24 reference assembly for *H. mississippiense* strains. Coverage per base was normalized based on median coverage per base between the genes SRE1 and MET1, Chr3 bases 2533970 to 3746857 for UCSF3 and the syntenic region on Chr2 for assembly WU24, 2400021 to 2941628. Strains sequenced from in-lab experimentation were generated directly from single colonies and had distinct nearly 1× or 2× coverage throughout nonrepetitive chromosomal regions. For these isolates, coverage through the critical region determined ploidy categorization. One boundary of the critical region was defined by the CNV region in two strains derived by CRISPR deletion of URA5 from G217B. These two strains retain hyphal bias despite having a very limited region of Chr7 duplication. The other boundary was determined by the CNV in four yeast-biased strains that have duplication of only the left-hand portion of Chr7. These four strains were selected for yeast bias versus their ancestral aneuploid strain, a hyphal-biased strain containing fluorescent reporters. The critical region was found to be Chr7 bases 1466188 to 1800070 in assembly UCSF3 and Chr6 bases 234820 to 597144 in assembly WU24. For population isolates, which included a few with intermediate coverage levels, 1.25× median coverage in the critical region was used as a threshold to determine ploidy categorization.

Assessment of the rate of aneuploidy gain and loss

For determination of colony morphology bias conversion rate, proliferation from a single parental cell was counted by hemocytometer through growth in colonies and brief growth in liquid culture for an average of 31 doublings total prior to the assessment of progeny morphology. To determine aneuploid loss rates, a total of 11,364 colonies were categorized for morphology with 44 colonies switching in morphology among 6 experimental replicates (replicates averaged 32 doublings). To determine aneuploid gain rates, a total of 9,750 colonies were categorized for morphology with 19 colonies switching in morphology among 5 experimental replicates (replicates averaged 31 doublings). In these rate calculations, the few sectored colonies (e.g., colony adjacent to green star in Fig 1B) were categorized by the dominant morphology of the colony.

Conditions for competition assays

To obtain matched steady-state yeast and hyphae, cultures were started from single yeast colonies and grown as yeast for two days. These starter cultures were used to inoculate plates without GlcNAc at 37°C and with GlcNAc at 25°C which were grown in parallel for 20 days. Hyphal cultures were then started from 25°C plates, grown for 2 days in liquid HMM supplemented with GlcNAc to ensure full hyphal conversion, passaged 1:6 in HMM without GlcNAc for five days, full hyphal growth was confirmed by microscopy, and hyphae were passaged 1:25 in media without GlcNAc and grown for seven days. Yeast cultures were started from 37°C plates and passaged 1:25 3 times a week in media without GlcNAc for the same two weeks as the hyphal cultures. After 2 weeks of steady-state liquid growth, competition mixtures were made. Yeast were mixed at equal OD_{600} (where appropriate) and diluted to OD_{600} of 0.2, and hyphae were mixed to equal volume (where appropriate) and total dilution of 1:25. For steady-state yeast competition, cells stayed in standard yeast conditions with passage three times a week. For morphology transitions, yeast were then brought to standard hyphal conditions and hyphae were brought to standard yeast conditions on day 0, and cultures were passaged 1:25 every seven days throughout the competition, at which points samples for DNA sequencing and microscopy were taken as described above. Media was not supplemented with GlcNAc during these transitions.

For competition experiments, at least four microscopy images were taken per flask per time point. All images were combined and randomized, with 25 repeating images to ensure consistency, then categorized by morphology. Images

were randomly shown and categorized based on an image key as all yeast (1), vast majority yeast (2), mixed (3), vast majority hyphae (4), all hyphae (5) (S3 Fig). Each of the 25 images shown twice was scored the same way both times. The mean image score was taken as the score for each sample time point, and the mean and standard deviation among replicate sample scores is plotted.

In competition assays, two barcodes, defined as the alleles at two non-coding SNPs, were used to identify the starting strains as either euploid or aneuploid. Each barcode was assessed for either euploid or aneuploid starting cells to ensure that the SNP barcode itself did not affect the data. Fraction aneuploid barcode was calculated at each SNP as (aneuploid barcode allele counts)/(total counts for both alleles) and the mean over the two locations is reported. Chr7 copy number was calculated based on median coverage in Chr7 versus median coverage in the normalizer region.

Assessment of survival and fungal burden in murine model

Murine infections were performed as previously described [35] using 8–12 week-old female C57Bl/6 (Jackson Laboratories Strain 000664) mice infected intranasally with 1.0×10^6 WU15 + *URA5* yeast per mouse. For survival analysis, mice were monitored daily for both symptoms of disease (hunching, panting, ears tucked back) and weight loss (weight loss relative to day 0) as part of our euthanasia criteria over the course of 21 days. Mice were euthanized after they exhibited 3 days of sustained weight loss to ≤75% of their maximum weight in addition to one other symptom. To determine fungal burden and to count and categorize colonies of each morphology bias, lungs and spleens were collected from infected mice and homogenized in PBS. Homogenates were sonicated, diluted, plated on HMM and grown at 37°C then at 25°C for 8–13 days, at which point colonies with each phenotype were counted as previously described.

RNA sequencing

Steady-state and transitioned cultures were prepared as for competition assays. Steady state and two-day transition timepoints were taken from two-day-old cultures. Seven-day transition cultures were collected after seven days in conditions inducing the opposite morphology without passaging. Each condition was assessed in biological triplicate; media was not supplemented with GlcNAc for any of these conditions. *HYF1* increased copy number, *HYF2* increased copy number, and control samples for RNA sequencing were collected from cultures started at OD_{600} of 0.2 then grown for 2 days in standard yeast conditions in HMM media not supplemented with GlcNAc, the same as was done for aneuploid and euploid steady state yeast included in the RNA sequencing transition assays. P_{ACT1}-*HYF1* and control empty vector samples for RNA sequencing were collected from yeast prepared the same way, grown for the final two days at either 37°C or 25°C, the same as was done for two conditions in the aneuploid and euploid RNA sequencing transition assays. All cells for RNA sequencing were collected by filtration, flash frozen in liquid nitrogen, and stored at −80°C prior to RNA extraction. RNA extraction was performed as previously described [21]. mRNA isolation, and RNAseq library preparation were performed as previously described [21] other than for comparison of P_{ACT1}-*HYF1* and control empty vector which was performed by SeqCenter, LLC (Pittsburgh, PA). Average fragment size and presence of excess adapter was determined with High Sensitivity DNA Bioanalyzer chip from Agilent Technologies (Santa Clara, CA). Library concentration was measured by Qubit dsDNA BR Quantification Assay Kit (Invitrogen) then pooled with equal DNA mass from each library. The final pooled libraries were submitted to the UCSF Center for Advanced Technology for sequencing on an Illumina Novaseq X 10B sequencer.

RNA sequencing data analysis

Transcript abundances were quantified based on version ucsf_hc.01_1.G217B of the *Histoplasma* G217B transcriptome (S5 Data of Gilmore and colleagues [20]). Relative abundances (reported as TPM values [73]) and estimated counts (est_counts) of each transcript in each sample were estimated by alignment-free comparison of k-mers between the reads and mRNA sequences using KALLISTO version 0.46.2 [74]. Further analysis was restricted to transcripts with estimated

counts ≥ 10 in at least three samples. Differentially expressed genes were identified by comparing replicate means for contrasts of interest using LIMMA version 3.46.0 [75,76]. Genes were considered significantly differentially expressed if they were statistically significant (at 5% FDR) with an effect size of at least 1.5× (absolute $\log_2$ fold change ≥ 0.585) for a given contrast.

Comparative transcriptome analysis

For fungi with RNAseq data available for hyphal and yeast (or spherule, for Coccidioides) (S5 Table), reads were downloaded from the SRA. Corresponding transcriptome sequences were downloaded from the sources indicated in S5 Table and indexed for KALLISTO. Transcripts were quantified for each sample with KALLISTO version 0.46.2 [74], invoked as:

kallisto quant -i TRANSCRIPTOME.idx EXTRA RUNS

where TRANSCRIPTOME.idx is the appropriate index file, RUNS are gzipped FASTQ files for all runs corresponding to the given sample, and EXTRA are additional flags for appropriate experiment-specific handling of single-ended and strand-specific reads, as given in the "extra flags" column of S5 Table. Further analysis was restricted to transcripts with ≥10 counts in at least half of the samples from a given dataset. Yeast/hyphal or spherule/hyphal contrasts and BH adjusted *p*-values were estimated using LIMMA version 3.46.0 [75,76] and genes were considered significantly differentially expressed if they were statistically significant (at 5% FDR) with an effect size of at least 2× (absolute $\log_2$ fold change ≥ 1). *Histoplasma* ortholog groups were taken from S13 Data of Gilmore and colleagues [20]. Orthologous genes between G217B and the remaining species were determined by InParanoid version 1.35 [77]. We used Fisher's exact test with BH multiple hypothesis correction to test for significant enrichment of hyphal-enriched orthologs from the comparison transcriptomes in the set of 144 HYF1 + aneuploid-enriched genes. Kernel density estimates of these distributions are plotted in Fig 7G using the gaussian_kde function from SciPy [78].

Generation of increased copy number and constitutive expression strains

To construct *Histoplasma* strains with increased copy number of specific regions (regions indicated by black bars in S6A–S6E and S7D Figs), these regions were amplified from extracted genomic DNA (S2 Table). Regions were then cloned into a *Histoplasma* entry vector containing telomere repeats and the gene *URA5*. Transformation of these linearized plasmids into *Histoplasma* was performed as previously described [79] into WU15-based strains. Strains containing ectopic plasmids with native regulatory elements were used for RNA sequencing shown in Fig 7. For *HYF1* and *HYF2*, constitutive expression plasmids were similarly made by amplifying the region indicated in gray in S6D and S7D Figs which were cloned into a *Histoplasma* entry vector with the *ACT1* promoter.

Other analysis and statistics

Venn diagrams were made using venn3, except the diagram in 7A which was made using eulerr. Heatmaps were generated in Java TreeView 1.1.6r4 [80]. Survival curve was generated using Prism. Throughout this paper, *p*-values equal to or above 0.05 are considered not significant, *p*-values < 0.05 are indicated by *, *p*-values < 0.01 are indicated by **, *p*-values < 0.001 are indicated by ***. Specific statistical tests are noted in figure legends and methods.

Supporting information

S1 Table. Strains used.
(TXT)

S2 Table. Primers used for cloning.
(TXT)

S3 Table. KALLISTO estimated counts and LIMMA-fit values for time course expression profiles. Excel-compatible tab-delimited text conforming to JavaTreeView extended CDT format. Each row is a transcript, with the UNIQID column giving the ucsf_hc.01_1.G217B systematic gene name. The NAME column gives short names taken from Data S1 of Voorhies, 2022 [70] with additional names and corrections based on human curation. The next 36 columns give KALLISTO estimated counts for each transcript in each sample. The next columns give LIMMA BH-adjusted p-values for differential expression in each of the 20 contrasts, and the corresponding LIMMA-fit log2 ratios are given in the final 20 columns. Description and HcG217B_pred (WUSTL G217B predicted gene accessions), HcG217B_rc (Edwards and colleagues. G217B transcript accessions [44]), and HcG217B_acc (UCSF1 gene accessions [70]); and Ryp1_ChIP, Ryp2_ChIP, Ryp3_ChIP, and Ryp4_C (indicating genes with promoters bound by Ryp TFs in Beyhan, 2013 [18]) are taken from Data S1 of Voorhies, 2022 [70]. UCSF2_acc and UCSF3_acc give gene accessions from the G217B assemblies of Heater, 2025 [24]. CR is "critical_region" for genes in the critical region, "chr7" for genes on chromosome 7 but outside of the critical region, and blank for all other genes. GWEIGHT is a placeholder column for JavaTreeView compatibility. The estimated counts in this file are sufficient to recapitulate the LIMMA analysis.
(TXT)

S4 Table. KALLISTO estimated counts and LIMMA-fit values for expression profiles of strains with elevated HYF1 or HYF2 copy number. Excel-compatible tab-delimited text conforming to JavaTreeView extended CDT format. Columns are as in table for time course expression profiles, except that the count, *p*-value, and log2 ratio columns correspond to the HYF1 and HYF2 elevated expression experiment. The estimated counts in this file are sufficient to recapitulate the LIMMA analysis.
(TXT)

S5 Table. Data sources for comparative transcriptome analysis. Excel-compatible tab-delimited text. Each row corresponds to a profile plotted in Fig 7G. Columns give a shorthand name, taxonomic details (genus, species, strain), data accessions (GEO and SRA) and reference (PubMed PMID), sequencing layout (paired or single), extra flags supplied to KALLISTO (see Materials and methods), the specific SRA sample accessions used for the yeast or hyphae expression profiles, and the source (URL or GenBank accession) of the transcriptome sequences used to build the KALLISTO index.
(TXT)

S6 Table. Comparative transcriptome profiles. Transcriptome profiles based on data in the sources indicated in S5 Table. Excel-compatible tab-delimited text conforming to JavaTreeView extended CDT format. UNIQID, NAME, Description, and annotation columns are as for S3 Table. Remaining columns, distinguished by the shorthand names from S5 Table, give ortholog gene IDs, LIMMA estimated log ratios, and LIMMA *p*-values for each comparison dataset.
(TXT)

S7 Table. Comparative transcriptome profiles for genes up in *HYF1* and aneuploid. Excel-compatible tab-delimited text conforming to JavaTreeView extended CDT format. Identical to S6 Table, but restricted to genes up in *HYF1* and aneuploid (green curves in Fig 7G).
(TXT)

S8 Table. KALLISTO estimated counts and LIMMA-fit values for expression profiles of strains carrying P_{ACT1}-*HYF1* plasmid or vector control. Excel-compatible tab-delimited text conforming to JavaTreeView extended CDT format. Columns are as in S3 Table (time course expression profiles), except that the count, *p*-value, and log2 ratio columns correspond to the *HYF1* constitutive expression experiment. The estimated counts in this file are sufficient to recapitulate the LIMMA analysis.
(TXT)

S9 Table. Data used for the calculation of Chr7 gain and loss rates. Table in Excel-compatible tab-delimited text format. As is indicated by the first column, rows show parent identifiers (including the term "Aneuploid" or "Euploid"), the number of rough colonies counted, the number of smooth colonies counted, and the doublings that separate parent and progeny.
(TXT)

S10 Table. Passage and competitions of yeast or hyphal strains differing in chromosome copy number in yeast- or hyphal-inducing conditions. Excel-compatible tab-delimited text file giving per sample phenotypes derived from sequencing and microscopy of passaged strains. Columns give sequenced sample name, parental source, colony morphology of parental (or mix for 1:1 mixture of rough:smooth), initial (Y)east or (H)yphal morphology of innoculum, growth temperature (or "first" for $t = 0$), day of sample collection, chr7 CNV and parental ratio inferred from sequencing, and morphology score from microscopy.
(TXT)

S11 Table. Survival of mice infected with aneuploid and/or euploid *Histoplasma*. Excel-compatible comma-separated-values table. First column gives mouse identifier. Second column gives time point. Remaining columns indicate infection type: euploid (ND), 1:1 aneuploid:euploid mixture (Mix), aneuploid (Dup), or mock infection (Mock) and are marked either 1 for time of euthanization or 0 for mice surviving through day 21 of the experiment.
(CSV)

S12 Table. Fungal burden in mice infected with aneuploid and/or euploid *Histoplasma.* Table in Excel-compatible tab-delimited text format. Each set of five columns provides data for a different condition: day 0 lung at hour 4 followed by day 7 lung followed by day 7 spleen. Within each set of five, the columns show inoculum ploidy, mouse identifier, dilution factor, smooth cells counted, rough cells counted. Each set is separated by an empty column for clarity. For each organ homogenate, two dilutions were plated, each in duplicate. Data from each plate is shown in a separate row, and "na" indicates a plate that was not counted. For day 7 plates, colonies were counted for half of the area of each plate. Organs were homogenized in 1 ml of PBS and 0.1 ml diluted homogenate was plated.
(TXT)

S1 Code. Code for quantification of passaged strains. ZIP archive of PYTHON modules, scripts, and JUPYTER notebooks for analysis of the experiments in Fig 3. The code is documented in a README html file at the top level of the archive.
(ZIP)

S1 Fig. A) Microscopy of strains at 37°C and after 6 days at 25°C. A population of wild-type *Histoplasma* spontaneously obtained an intermediate morphological phenotype. Ten random single colonies were isolated from this population, three of which were found to have a hyphal-bias and seven of which were found to have a yeast-bias. **B)** Four representative isolates are shown. These 10 isolates were subsequently sequenced, and a perfect correlation between ploidy and morphology bias was found, as shown in the first row of data in C. **C)** Summary of the sources for 64 strains for which paired aneuploid and euploid strains were derived from a parental source (64 strains shown in Fig 1D). For these strains, at least one matched strain was generated at the same time, from the same parental source, but of the opposite morphology bias. The first row is described in S1A and S1B. For subsequent rows, colony morphology-bias was used to isolate yeast-biased and hyphal-biased progeny of aneuploid cells, euploid cells, and cells from a mouse lung. **D)** Sources for the 13 strains that were included in the analysis in Fig 1C. These include strains ancestral to some of those shown above, as well as strain sets used to define a boundary of the critical region (indicated by asterisks) and controls. A total of 77 strains were phenotyped then subsequently genotyped for this analysis. Fisher's exact *p*-value was $< 1 \times 10^{-7}$ for correspondence between morphology bias and critical region coverage. More information on strains is available in S1 Table.
(EPS)

S2 Fig. Heatmaps showing copy number (CN) variation in natural isolates (clinical isolates and a limited number of available environmental population isolates) of *Histoplasma ohiense* (A) *Histoplasma mississippiense* (B), and other *Histoplasma* (C). The number of isolates in each category is listed parenthetically on the y-axis. The *H. ohiense* reference genome is used for other *Histoplasma* isolates shown in C. For each CNV heatmap, the critical region for each genome is highlighted in orange. Chromosomes are indicated, along with location of rDNA (R). **D)** Optical density (OD_{600}) growth curve of aneuploid and euploid yeast grown in standard yeast liquid growth conditions over the course of 141.5 hours. Fill indicates standard deviation at each point. **E)** Plots showing copy number variation in strains selected for sequencing from aneuploid gain and loss rate experiments, which are also included in the counts in Figs 1C, 1D, and S1. The data underlying this Figure can be found in S9 Table.
(EPS)

S3 Fig. Legend used for scoring of microscopy images. Legend shows 1: all yeast, 2: vast majority yeast, 3: mix, 4: vast majority hyphae, 5: all hyphae. Images with few cells or unclear images were not given a numeric score.
(EPS)

S4 Fig. Competition of aneuploid and euploid yeast grown continuously for four weeks. Plot shows fraction of aneuploid starting strain barcode from full genome sequencing. Fill indicates standard deviation at each point. The data underlying this Figure can be found in S10 Table.
(EPS)

S5 Fig. A, B) Distribution of log ratios for expression in aneuploid versus euploid cells as in Fig 5 showing (from top to bottom) steady state yeast, a 2-day transition to hyphae, a 7-day transition to hyphae, steady state hyphae, a 2-day transition to yeast, and a 7-day transition to yeast. Distributions of genes in chromosome 7 are shown in blue in A, HPS and YPS gene distributions are shown in B. **C)** Heatmap with the same columns as in Fig 5I–5L showing all predicted genes. The first two columns show transcript abundance in hyphae/yeast (column 1: euploid, column 2: aneuploid) (yellow versus red). Columns 3–8 show transcript abundance in aneuploid/euploid cells through yeast-to-hyphal transition (37–25°C) and hyphal to yeast transition (25–37°C) (green versus magenta). For each time point of each transition (steady state, day 2, and day 7), fit for each plotted transcript is derived from 3 replicates. Final column indicates transcripts encoded by genes on Chr7 in light blue. Genes are sorted based on the ratio of the first and third data columns, as noted by arrowheads below heatmap. (Specifically, the sort is on the element-wise arctangent of (euploid hyphae/yeast)/(yeast aneuploid/euploid) as implemented in arctan2 from numpy). Approximate locations of genes of interest are noted to the right of this heatmap. **D)** Heatmap with the same columns as S5C, showing genes that have been shown to affect mouse survival. **E)** Heatmap with similar columns as S5C showing previously defined set of additional virulence factors. None of these genes are on Chr7, so the ninth column is not included in this subfigure. **F)** Number of genes significantly enriched in yeast (YSG, red) or hyphae (HSG, yellow) or with context-dependent enrichment (other, dark gray, *e.g.,* YSG in aneuploid but HSG in euploid) or not significantly differential in the hyphae vs. yeast comparison (neutral, light gray) stacked. Genes are divided into separate bars based on ploidy-dependent differential expression in yeast, hyphae, both, or neither (ploidy-independent). The full set of plots accounts for all 11,795 analyzed genes, with each gene counted in exactly one bar. The data underlying this Figure can be found in S3 Table.
(EPS)

S6 Fig. A–F) Locations chosen for regions of the critical region added on ectopic plasmids (black bars). Each plot in A–F shows ribosomal footprinting [20] in yeast (red) and hyphae (olive) below RNA-seq in yeast (red) and hyphae (olive) followed by locations of predicted genes (gray) then *RYP1–4* ChIP enrichment in (red, green, blue, and purple, respectively) [18]. For each plot, primary gene is labeled above plot and shown in darker gray fill while adjacent upstream gene is labeled below plot and indicated by black line in predicted gene subplot. **D)** The region used for constitutive

expression of *HYF1* (under the control of the ACT1 promoter) is also indicated by gray bar. Y-axis limits are consistent in A–E. **F)** Multiple alignment of *HYF1* and orthologs, colored by conservation and annotated as in S1 Fig of Odenbach and colleagues [71].
(EPS)

S7 Fig. A) Location of HYF2 and complementation region as shown for other genes in Fig 6A. **B)** Microscopy of cells with increased copy number of *HYF2* as shown for other genes in Fig 6B. **C)** Microscopy of cells with constitutive expression ("CE") of *HYF2* driven by the *ACT1* promoter as shown for *HYF1* in Fig 6C. Experiments were performed simultaneously with those shown in Fig 6 and thus share control images. **D)** Locations of regions used for increased copy number of *HYF2* and constitutive expression of *HYF2* by the *ACT1* promoter as shown for other genes in S6A–S6E Fig. Y-axis limits are the same as in S6A–S6E Fig except the ChIP track which has a maximum *y* value of twice that shown in S6 Fig to show full range. **E)** Heatmap showing all predicted transcripts with the same columns as in Fig 5 in addition to a column showing transcript abundance in *HYF2* increased copy number yeast over yeast containing a control plasmid. Transcripts are sorted based on the first column (aneuploid/euploid). **F–K)** Plots showing transcriptomic data for *HYF2* as shown for *HYF1* in Fig 7A–7F. Note for H and I, the y-axis change in scale used to show the more subtle effects of *HYF2*. **L)** Multiple alignment of *HYF2* and orthologs, colored by conservation. The data underlying this Figure can be found in S4 Table.
(EPS)

S8 Fig. Constitutive expression of *HYF1* at 37°C induces a persistent hyphal-promoting regulon. A) TMM-normalized kallisto estimates of transcript abundance of *HYF1* in units of $\log_2$(cpm). Blue circles indicate individual replicates, and black lines indicate replicate means. **B, C)** Distribution of relative transcript abundance in P_{ACT1}-*HYF1* strains versus control strains at $t = 0$, 37°C (B) or $t = 2$d, RT (C) for all *Histoplasma* genes (gray) or genes from the "up in *HYF1* and aneuploid" regulon defined in Fig 7G (green). Asterisks define significance using the same criteria as Fig 7G. The data underlying this Figure can be found in S8 Table.
(EPS)

Acknowledgments

We thank Daniel Matute, William Goldman, and Victoria Sepulveda for generously sharing clinical *Histoplasma* isolates. We thank Praise Oo for initial growth and assessment of these clinical isolates. We thank Sandy Johnson, Carol Gross, Joe Bondy-Denomy, and Suzanne Noble for useful suggestions. We thank members of the Sil and Noble labs for helpful discussions. The UCSF Center for Advanced Technology and Biohub-San Francisco supported this research through use of equipment and/or sequencing. AS is a Biohub, San Francisco, investigator.

Author contributions

Conceptualization: Sarah Heater, Mark Voorhies, Anita Sil.

Data curation: Sarah Heater, Rosa A. Rodriguez, Bevin C. English.

Formal analysis: Sarah Heater, Mark Voorhies, Rosa A. Rodriguez, Bevin C. English.

Funding acquisition: Anita Sil.

Investigation: Sarah Heater, Rosa A. Rodriguez.

Methodology: Sarah Heater, Mark Voorhies, Bevin C. English.

Resources: Sarah Heater.

Software: Sarah Heater, Mark Voorhies.

Validation: Sarah Heater, Mark Voorhies.

Visualization: Sarah Heater, Mark Voorhies.

Writing – original draft: Sarah Heater.

Writing – review & editing: Sarah Heater, Mark Voorhies, Rosa A. Rodriguez, Bevin C. English, Anita Sil.

References

1. Kakade P, Sircaik S, Maufrais C, Ene IV, Bennett RJ. Aneuploidy and gene dosage regulate filamentation and host colonization by *Candida albicans*. Proc Natl Acad Sci U S A. 2023;120(11):e2218163120. https://doi.org/10.1073/pnas.2218163120 PMID: 36893271
2. Zhou X, Hilk A, Solis NV, Scott N, Beach A, Soisangwan N, et al. Single-cell detection of copy number changes reveals dynamic mechanisms of adaptation to antifungals in *Candida albicans*. Nat Microbiol. 2024;9(11):2923–38. https://doi.org/10.1038/s41564-024-01795-7 PMID: 39227665
3. Hull RM, Cruz C, Jack CV, Houseley J. Environmental change drives accelerated adaptation through stimulated copy number variation. PLoS Biol. 2017;15(6):e2001333. https://doi.org/10.1371/journal.pbio.2001333 PMID: 28654659
4. Chen G, Bradford WD, Seidel CW, Li R. Hsp90 stress potentiates rapid cellular adaptation through induction of aneuploidy. Nature. 2012;482(7384):246–50. https://doi.org/10.1038/nature10795 PMID: 22286062
5. Loll-Krippleber R, Feri A, Nguyen M, Maufrais C, Yansouni J, d'Enfert C, et al. A FACS-optimized screen identifies regulators of genome stability in *Candida albicans*. Eukaryot Cell. 2015;14(3):311–22. https://doi.org/10.1128/EC.00286-14 PMID: 25595446
6. Vande Zande P, Zhou X, Selmecki A. The dynamic fungal genome: polyploidy, aneuploidy and copy number variation in response to stress. Annu Rev Microbiol. 2023;77:341–61. https://doi.org/10.1146/annurev-micro-041320-112443 PMID: 37307856
7. Gugnani HC, Denning DW. Infection of bats with *Histoplasma* species. Med Mycol. 2023;61(8):myad080. https://doi.org/10.1093/mmy/myad080 PMID: 37553137
8. Taylor JW, Barker BM. The endozoan, small-mammal reservoir hypothesis and the life cycle of *Coccidioides* species. Med Mycol. 2019;57(Supplement_1):S16–20. https://doi.org/10.1093/mmy/myy039 PMID: 30690603
9. WHO fungal priority pathogens list to guide research, development and public health action. Available from: https://www.who.int/publications/i/item/9789240060241
10. Moreira LM, et al. Molecular detection of *Histoplasma capsulatum* in Antarctica. Emerg Infect Dis. 2022;28:2100–4.
11. Manos NE, Ferebee SH, Kerschbaum WF. Geographic variation in the prevalence of histoplasmin sensitivity. Dis Chest. 1956;29(6):649–68. https://doi.org/10.1378/chest.29.6.649 PMID: 13317782
12. Edwards LB, Acquaviva FA, Livesay VT, Cross FW, Palmer CE. An atlas of sensitivity to tuberculin, PPD-B, and histoplasmin in the United States. Am Rev Respir Dis. 1969;99(4):Suppl:1-132. PMID: 5767603
13. Adenis AA, Valdes A, Cropet C, McCotter OZ, Derado G, Couppie P, et al. Burden of HIV-associated histoplasmosis compared with tuberculosis in Latin America: a modelling study. Lancet Infect Dis. 2018;18(10):1150–9. https://doi.org/10.1016/S1473-3099(18)30354-2 PMID: 30146320
14. Pulido-Camarillo E, Sahaza JH, de Souza Pitangui N, Mendes-Giannini MJS, Fusco-Almeida AM, Pérez-Torres A, et al. Unusual differences in the pulmonary histopathology of mice after intranasal infection with mycelial propagules of *Histoplasma capsulatum* strains classified as LAm A2 and NAm 2 *Phylogenetic* Species. J Fungi (Basel). 2023;9(10):974. https://doi.org/10.3390/jof9100974 PMID: 37888230
15. Sepúlveda VE, Rader JA, Li J, Goldman WE, Matute DR. Phenotypic characterization of cryptic species in the fungal pathogen *Histoplasma*. mSphere. 2024;9:e00009-24.
16. Nemecek JC, Wüthrich M, Klein BS. Global control of dimorphism and virulence in fungi. Science. 2006;312(5773):583–8. https://doi.org/10.1126/science.1124105 PMID: 16645097
17. Nguyen VQ, Sil A. Temperature-induced switch to the pathogenic yeast form of *Histoplasma capsulatum* requires Ryp1, a conserved transcriptional regulator. Proc Natl Acad Sci U S A. 2008;105(12):4880–5. https://doi.org/10.1073/pnas.0710448105 PMID: 18339808
18. Beyhan S, Gutierrez M, Voorhies M, Sil A. A temperature-responsive network links cell shape and virulence traits in a primary fungal pathogen. PLoS Biol. 2013;11(7):e1001614. https://doi.org/10.1371/journal.pbio.1001614 PMID: 23935449
19. Webster RH, Sil A. Conserved factors Ryp2 and Ryp3 control cell morphology and infectious spore formation in the fungal pathogen *Histoplasma capsulatum*. Proc Natl Acad Sci U S A. 2008;105(38):14573–8. https://doi.org/10.1073/pnas.0806221105 PMID: 18791067
20. Gilmore SA, Voorhies M, Gebhart D, Sil A. Genome-wide reprogramming of transcript architecture by temperature specifies the developmental states of the human pathogen *Histoplasma*. PLoS Genet. 2015;11(7):e1005395. https://doi.org/10.1371/journal.pgen.1005395 PMID: 26177267
21. Assa D, Voorhies M, Sil A. Chemical stimuli override a temperature-dependent morphological program by reprogramming the transcriptome of a fungal pathogen. mBio. 2025;16(10):e0223425. https://doi.org/10.1128/mbio.02234-25 PMID: 40928299
22. Rodriguez L, Voorhies M, Gilmore S, Beyhan S, Myint A, Sil A. Retraction: opposing signaling pathways regulate morphology in response to temperature in the fungal pathogen *Histoplasma capsulatum*. PLoS Biol. 2023;21(3):e3002060. https://doi.org/10.1371/journal.pbio.3002060 PMID: 36944162
23. Rodriguez L, Voorhies M, Gilmore S, Beyhan S, Myint A, Sil A. Opposing signaling pathways regulate morphology in response to temperature in the fungal pathogen *Histoplasma capsulatum*. PLoS Biol. 2019;17(9):e3000168. https://doi.org/10.1371/journal.pbio.3000168 PMID: 31568523

24. Heater S, Voorhies M, Sil A. Genome dynamics and chromosome structural variations in *Histoplasma ohiense* , a fungal pathogen of humans. Cold Spring Harbor Laboratory; 2025. https://doi.org/10.1101/2025.05.05.652209
25. Sepúlveda VE, Márquez R, Turissini DA, Goldman WE, Matute DR. Genome sequences reveal cryptic speciation in the human pathogen *Histoplasma capsulatum*. mBio. 2017;8(6):e01339-17. https://doi.org/10.1128/mBio.01339-17 PMID: 29208741
26. Bagal UR, Gade L, Benedict K, Howell V, Christophe N, Gibbons-Burgener S, et al. A Phylogeographic description of *Histoplasma capsulatum* in the United States. J Fungi (Basel). 2023;9(9):884. https://doi.org/10.3390/jof9090884 PMID: 37754992
27. Tenório BG, Kollath DR, Gade L, Litvintseva AP, Chiller T, Jenness JS, et al. Tracing histoplasmosis genomic epidemiology and species occurrence across the USA. Emerg Microbes Infect. 2024;13(1):2315960. https://doi.org/10.1080/22221751.2024.2315960 PMID: 38465644
28. Sharp NP, Sandell L, James CG, Otto SP. The genome-wide rate and spectrum of spontaneous mutations differ between haploid and diploid yeast. Proc Natl Acad Sci U S A. 2018;115(22):E5046–55. https://doi.org/10.1073/pnas.1801040115 PMID: 29760081
29. Zhu YO, Siegal ML, Hall DW, Petrov DA. Precise estimates of mutation rate and spectrum in yeast. Proc Natl Acad Sci U S A. 2014;111(22):E2310-8. https://doi.org/10.1073/pnas.1323011111 PMID: 24847077
30. Sui Y, et al. Genome-wide mapping of spontaneous genetic alterations in diploid yeast cells. Proc Natl Acad Sci U S A. 2020;117:28191.
31. Homer CM, Voorhies M, Walcott K, Ochoa E, Sil A. Transcriptomic atlas throughout *Coccidioides* development reveals key phase-enriched transcripts of this important fungal pathogen. PLoS Biol. 2025;23(4):e3003066. https://doi.org/10.1371/journal.pbio.3003066 PMID: 40233121
32. Isaac DT, Berkes CA, English BC, Murray DH, Lee YN, Coady A, et al. Macrophage cell death and transcriptional response are actively triggered by the fungal virulence factor Cbp1 during *H. capsulatum* infection. Mol Microbiol. 2015;98(5):910–29. https://doi.org/10.1111/mmi.13168 PMID: 26288377
33. Azimova D, Herrera N, Duvenage L, Voorhies M, Rodriguez RA, English BC, et al. Cbp1, a fungal virulence factor under positive selection, forms an effector complex that drives macrophage lysis. PLoS Pathog. 2022;18(6):e1010417. https://doi.org/10.1371/journal.ppat.1010417 PMID: 35731824
34. Youseff BH, Holbrook ED, Smolnycki KA, Rappleye CA. Extracellular superoxide dismutase protects *Histoplasma* yeast cells from host-derived oxidative stress. PLoS Pathog. 2012;8(5):e1002713. https://doi.org/10.1371/journal.ppat.1002713 PMID: 22615571
35. Rodriguez RA, Azimova D, Voorhies M, English BC, Symington J, Sil A. Expansion of secreted cystine knot proteins reveals virulence factors in the human fungal pathogen *Histoplasma*. Cell Rep. 2025;44(11):116465. https://doi.org/10.1016/j.celrep.2025.116465 PMID: 41138184
36. Hu S-J, Zheng H, Li X-P, Li Z-X, Xu C, Li J, et al. Ada2 and Ada3 regulate hyphal growth, asexual development, and pathogenicity in *Beauveria bassiana* by maintaining Gcn5 acetyltransferase activity. Microbiol Spectr. 2023;11(3):e0028123. https://doi.org/10.1128/spectrum.00281-23 PMID: 37052485
37. Pukkila-Worley R, Peleg AY, Tampakakis E, Mylonakis E. Candida albicans hyphal formation and virulence assessed using a *Caenorhabditis elegans* infection model. Eukaryot Cell. 2009;8(11):1750–8. https://doi.org/10.1128/EC.00163-09 PMID: 19666778
38. Goyard S, Knechtle P, Chauvel M, Mallet A, Prévost M-C, Proux C, et al. The Yak1 kinase is involved in the initiation and maintenance of hyphal growth in *Candida albicans*. Mol Biol Cell. 2008;19(5):2251–66. https://doi.org/10.1091/mbc.e07-09-0960 PMID: 18321992
39. Cao H, Huang P, Zhang L, Shi Y, Sun D, Yan Y, et al. Characterization of 47 Cys2 -His2 zinc finger proteins required for the development and pathogenicity of the rice blast fungus *Magnaporthe oryzae*. New Phytol. 2016;211(3):1035–51. https://doi.org/10.1111/nph.13948 PMID: 27041000
40. Zhou S, Liu S, Guo C, Wei H, He Z, Liu Z, et al. The C2H2 Transcription factor Con7 regulates vegetative growth, cell wall integrity, oxidative stress, asexual sporulation, appressorium and hyphopodium formation, and pathogenicity in *Colletotrichum graminicola* and *Colletotrichum siamense*. J Fungi (Basel). 2024;10(7):495. https://doi.org/10.3390/jof10070495 PMID: 39057380
41. Shin S, Park J, Yang L, Kim H, Choi GJ, Lee Y-W, et al. Con7 is a key transcription regulator for conidiogenesis in the plant pathogenic fungus *Fusarium graminearum*. mSphere. 2024;9(5):e0081823. https://doi.org/10.1128/msphere.00818-23 PMID: 38591889
42. Lohse MB, Johnson AD. Identification and characterization of Wor4, a new transcriptional regulator of white-opaque switching. G3 (Bethesda). 2016;6(3):721–9. https://doi.org/10.1534/g3.115.024885 PMID: 26772749
43. Paul S, Bowyer P, Bromley M, Moye-Rowley WS. *Aspergillus fumigatus* ffmA encodes a C2H2-containing transcriptional regulator that modulates azole resistance and is required for normal growth. mSphere. 2022;7:e00938-21.
44. Edwards JA, Chen C, Kemski MM, Hu J, Mitchell TK, Rappleye CA. *Histoplasma* yeast and mycelial transcriptomes reveal pathogenic-phase and lineage-specific gene expression profiles. BMC Genomics. 2013;14:695. https://doi.org/10.1186/1471-2164-14-695 PMID: 24112604
45. Azadmanesh J, Gowen AM, Creger PE, Schafer ND, Blankenship JR. Filamentation involves two overlapping, but distinct, programs of filamentation in the pathogenic fungus *Candida albicans*. G3 (Bethesda). 2017;7(11):3797–808. https://doi.org/10.1534/g3.117.300224 PMID: 28951491
46. Muñoz JF, Gauthier GM, Desjardins CA, Gallo JE, Holder J, Sullivan TD, et al. The dynamic genome and transcriptome of the human fungal pathogen blastomyces and close relative emmonsia. PLoS Genet. 2015;11(10):e1005493. https://doi.org/10.1371/journal.pgen.1005493 PMID: 26439490
47. Yang E, Wang G, Woo PCY, Lau SKP, Chow W-N, Chong KTK, et al. Unraveling the molecular basis of temperature-dependent genetic regulation in *Penicillium marneffei*. Eukaryot Cell. 2013;12(9):1214–24. https://doi.org/10.1128/EC.00159-13 PMID: 23851338
48. Nigg M, Laroche J, Landry CR, Bernier L. RNAseq analysis highlights specific transcriptome signatures of yeast and mycelial growth phases in the Dutch elm disease fungus *Ophiostoma novo-ulmi*. G3 (Bethesda). 2015;5(11):2487–95. https://doi.org/10.1534/g3.115.021022 PMID: 26384770

49. Giosa D, Felice MR, Giuffrè L, Aiese Cigliano R, Paytuví-Gallart A, Lo Passo C, et al. Transcriptome-wide expression profiling of *Sporothrix schenckii* yeast and mycelial forms and the establishment of the Sporothrix Genome DataBase. Microb Genom. 2020;6(10):mgen000445. https://doi.org/10.1099/mgen.0.000445 PMID: 33034552

50. Kijpornyongpan T, Aime MC. Comparative transcriptomics reveal different mechanisms for hyphal growth across four plant-associated dimorphic fungi. Fungal Genet Biol. 2021;152:103565. https://doi.org/10.1016/j.fgb.2021.103565 PMID: 33991665

51. Han K-H, Kim JH, Moon H, Kim S, Lee S-S, Han D-M, et al. The *Aspergillus nidulans* esdC (early sexual development) gene is necessary for sexual development and is controlled by veA and a heterotrimeric G protein. Fungal Genet Biol. 2008;45(3):310–8. https://doi.org/10.1016/j.fgb.2007.09.008 PMID: 17977758

52. Fang W, St Leger RJ. RNA binding proteins mediate the ability of a fungus to adapt to the cold. Environ Microbiol. 2010;12(3):810–20. https://doi.org/10.1111/j.1462-2920.2009.02127.x PMID: 20050869

53. McFadden ER Jr, Pichurko BM, Bowman HF, Ingenito E, Burns S, Dowling N, et al. Thermal mapping of the airways in humans. J Appl Physiol (1985). 1985;58(2):564–70. https://doi.org/10.1152/jappl.1985.58.2.564 PMID: 3980358

54. Hutton JP, Durham JB, Miller DP, Everett ED. Hyphal forms of *Histoplasma capsulatum*. A common manifestation of intravascular infections. Arch Pathol Lab Med. 1985;109(4):330–2. PMID: 3885898

55. Svirbely JR, Ayers LW, Buesching WJ. Filamentous *Histoplasma capsulatum* endocarditis involving mitral and aortic valve porcine bioprostheses. Arch Pathol Lab Med. 1985;109(3):273–6. PMID: 3838457

56. Riddell J, et al. *Histoplasma capsulatum* endocarditis. Medicine (Baltimore). 2014;93:186–93.

57. Tian X, He G-J, Hu P, Chen L, Tao C, Cui Y-L, et al. *Cryptococcus neoformans* sexual reproduction is controlled by a quorum sensing peptide. Nat Microbiol. 2018;3(6):698–707. https://doi.org/10.1038/s41564-018-0160-4 PMID: 29784977

58. Décanis N, Tazi N, Correia A, Vilanova M, Rouabhia M. Farnesol, a fungal quorum-sensing molecule triggers *Candida albicans* morphological changes by downregulating the expression of different secreted aspartyl proteinase genes. Open Microbiol J. 2011;5:119–26. https://doi.org/10.2174/1874285801105010119 PMID: 22207890

59. Homer CM, Summers DK, Goranov AI, Clarke SC, Wiesner DL, Diedrich JK, et al. Intracellular action of a secreted peptide required for fungal virulence. Cell Host Microbe. 2016;19(6):849–64. https://doi.org/10.1016/j.chom.2016.05.001 PMID: 27212659

60. Gilmore SA, Naseem S, Konopka JB, Sil A. N-acetylglucosamine (GlcNAc) triggers a rapid, temperature-responsive morphogenetic program in thermally dimorphic fungi. PLoS Genet. 2013;9(9):e1003799. https://doi.org/10.1371/journal.pgen.1003799 PMID: 24068964

61. Gilchrist C, Stelkens R. Aneuploidy in yeast: segregation error or adaptation mechanism?. Yeast. 2019;36(9):525–39. https://doi.org/10.1002/yea.3427 PMID: 31199875

62. Nair J, Shetty S, Kasi CI, Thondehalmath N, Ganesh D, Bhat VR, et al. Preimplantation genetic testing for aneuploidy (PGT-A)-a single-center experience. J Assist Reprod Genet. 2022;39(3):729–38. https://doi.org/10.1007/s10815-022-02413-3 PMID: 35119550

63. Didion JP, Buus RJ, Naghashfar Z, Threadgill DW, Morse HC 3rd, de Villena FP-M. SNP array profiling of mouse cell lines identifies their strains of origin and reveals cross-contamination and widespread aneuploidy. BMC Genomics. 2014;15(1):847. https://doi.org/10.1186/1471-2164-15-847 PMID: 25277546

64. Rojas J, Hose J, Dutcher HA, Place M, Wolters JF, Hittinger CT, et al. Comparative modeling reveals the molecular determinants of aneuploidy fitness cost in a wild yeast model. Cell Genom. 2024;4(10):100656. https://doi.org/10.1016/j.xgen.2024.100656 PMID: 39317188

65. Jin X, Cheng AG, Chanin RB, Yu FB, Dimas A, Jasper M, et al. Comprehensive profiling of genomic invertons in defined gut microbial community reveals associations with intestinal colonization and surface adhesion. Microbiome. 2025;13(1):71. https://doi.org/10.1186/s40168-025-02052-7 PMID: 40059174

66. Lowrey LC, Kent LA, Rios BM, Ocasio AB, Cotter PA. An IS-mediated, RecA-dependent, bet-hedging strategy in *Burkholderia thailandensis*. Elife. 2023;12:e84327. https://doi.org/10.7554/eLife.84327 PMID: 36715687

67. Tan Z, Hays M, Cromie GA, Jeffery EW, Scott AC, Ahyong V, et al. Aneuploidy underlies a multicellular phenotypic switch. Proc Natl Acad Sci U S A. 2013;110(30):12367–72. https://doi.org/10.1073/pnas.1301047110 PMID: 23812752

68. Dumetz F, Imamura H, Sanders M, Seblova V, Myskova J, Pescher P, et al. Modulation of aneuploidy in *Leishmania donovani* during adaptation to different in vitro and in vivo environments and its impact on gene expression. mBio. 2017;8(3):e00599-17. https://doi.org/10.1128/mBio.00599-17 PMID: 28536289

69. van Gestel J, Koo B-M, Stürmer VS, Garriga-Canut M, Wagner J, Zanon A, et al. *Bacillus subtilis* in defense mode: switch-like adaptations to protistan predation. Proc Natl Acad Sci U S A. 2025;122(39):e2518989122. https://doi.org/10.1073/pnas.2518989122 PMID: 40991432

70. Voorhies M, Cohen S, Shea TP, Petrus S, Muñoz JF, Poplawski S, et al. Chromosome-level genome assembly of a human fungal pathogen reveals synteny among geographically distinct species. mBio. 2022;13(1):e0257421. https://doi.org/10.1128/mbio.02574-21 PMID: 35089059

71. Odenbach D, Breth B, Thines E, Weber RWS, Anke H, Foster AJ. The transcription factor Con7p is a central regulator of infection-related morphogenesis in the rice blast fungus *Magnaporthe grisea*. Mol Microbiol. 2007;64(2):293–307. https://doi.org/10.1111/j.1365-2958.2007.05643.x PMID: 17378924

72. Joehnk B, Ali N, Voorhies M, Walcott K, Sil A. Recyclable CRISPR/Cas9-mediated gene disruption and deletions in *Histoplasma*. mSphere. 2023;8:e00370-23.

73. Li B, Dewey CN. RSEM: accurate transcript quantification from RNA-Seq data with or without a reference genome. BMC Bioinformatics. 2011;12:323. https://doi.org/10.1186/1471-2105-12-323 PMID: 21816040

74. Bray NL, Pimentel H, Melsted P, Pachter L. Near-optimal probabilistic RNA-seq quantification. Nat Biotechnol. 2016;34(5):525–7. https://doi.org/10.1038/nbt.3519 PMID: 27043002

75. Smyth GK. Linear models and empirical bayes methods for assessing differential expression in microarray experiments. Stat Appl Genet Mol Biol. 2004;3:Article3. https://doi.org/10.2202/1544-6115.1027 PMID: 16646809

76. Ritchie ME, Phipson B, Wu D, Hu Y, Law CW, Shi W, et al. limma powers differential expression analyses for RNA-sequencing and microarray studies. Nucleic Acids Res. 2015;43(7):e47. https://doi.org/10.1093/nar/gkv007 PMID: 25605792

77. Remm M, Storm CE, Sonnhammer EL. Automatic clustering of orthologs and in-paralogs from pairwise species comparisons. J Mol Biol. 2001;314(5):1041–52. https://doi.org/10.1006/jmbi.2000.5197 PMID: 11743721

78. Virtanen P, Gommers R, Oliphant TE, Haberland M, Reddy T, Cournapeau D, et al. SciPy 1.0: fundamental algorithms for scientific computing in Python. Nat Methods. 2020;17(3):261–72. https://doi.org/10.1038/s41592-019-0686-2 PMID: 32015543

79. Woods JP, Heinecke EL, Goldman WE. Electrotransformation and expression of bacterial genes encoding hygromycin phosphotransferase and beta-galactosidase in the pathogenic fungus *Histoplasma capsulatum*. Infect Immun. 1998;66(4):1697–707. https://doi.org/10.1128/IAI.66.4.1697-1707.1998 PMID: 9529100

80. Saldanha AJ. Java Treeview—extensible visualization of microarray data. Bioinformatics. 2004;20(17):3246–8. https://doi.org/10.1093/bioinformatics/bth349 PMID: 15180930
